# Incidence of liver complications with hemochromatosis-associated *HFE* p.C282Y homozygosity: The role of central adiposity

**DOI:** 10.1097/HEP.0000000000001056

**Published:** 2024-08-23

**Authors:** Mitchell R. Lucas, Luke C. Pilling, Janice L. Atkins, David Melzer

**Affiliations:** Epidemiology and Public Health Group, Department of Clinical and Biomedical Sciences, Faculty of Health and Life Sciences, University of Exeter, Exeter, UK

**Keywords:** BMI, cirrhosis, diabetes, liver fibrosis, obesity

## Abstract

**Background and Aims:**

The *HFE* p.C282Y+/+ (homozygous) genotype and central adiposity both increase liver disease and diabetes risks, but the combined effects are unclear. We estimated waist-to-hip ratio (WHR) associations with incident clinical outcomes in routine care in p.C282Y+/+ participants in the UK Biobank community cohort.

**Approach and Results:**

Baseline WHR data available in 1297 male and 1602 female p.C282Y+/+ with 13.3-year mean follow-up for diagnoses. Spline regressions and Cox proportional hazard models were adjusted for age and genetic principal components. Cumulative incidence was from age 40 to 80 years. In p.C282Y+/+ males, there were positive linear WHR relationships for hospital inpatient–diagnosed liver fibrosis/cirrhosis (*p* = 2.4 × 10^−5^), liver cancer (*p* = 0.007), non-alcoholic fatty liver disease (*p* = 7.7 × 10^−11^), and type 2 diabetes (*p* = 5.1 × 10^−16^). The hazard ratio for high WHR in p.C282Y+/+ males (≥0.96; 33.9%) was 4.13 for liver fibrosis/cirrhosis (95% CI: 2.04–8.39, *p* = 8.4 × 10^−5^ vs. normal WHR); cumulative age 80 incidence 15.0% (95% CI: 9.8%–22.6%) versus 3.9% (95% CI: 1.9%–7.6%); for liver cancer, cumulative incidence was 9.2% (95% CI: 5.7%–14.6%) versus 3.6% (95% CI: 1.9%–6.6%). Hemochromatosis was diagnosed in 23 (96%) of the 24 high WHR p.C282Y+/+ males with incident fibrosis/cirrhosis. High WHR (≥0.85; 30.0%) p.C282Y+/+ females had raised hazards for liver fibrosis/cirrhosis (hazard ratio = 9.17, 95% CI: 2.51–33.50, *p* = 3.8 × 10^−7^) and Non-alcoholic fatty liver disease (hazard ratio = 5.17, 95% CI: 2.48–10.78, *p* = 1.2 × 10^−5^). Fibrosis/cirrhosis associations were similar in the subset with additional primary care diagnoses.

**Conclusions:**

In p.C282Y+/+ males and females, increasing WHR is associated with substantially higher risks of liver complications. Interventions to reduce central adiposity to improve these outcomes should be tested.

## Graphical abstract

**Figure:**
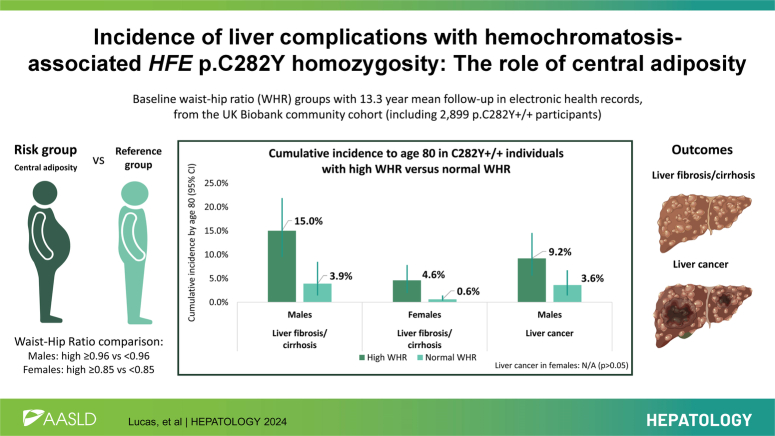

## INTRODUCTION

The iron overload condition, hemochromatosis, is the most common genetic disorder in people of Northern European ancestry.^1,2^ *HFE* gene mutations downregulate hepcidin protein, increasing circulating iron and, in some cases, causing iron accumulation.^3^ Iron overload can increase oxidative stress and inflammation in the liver, leading to insulin resistance, liver scarring, and hepatocellular carcinoma if left untreated.^4,5^

Among Northern Europeans, the *HFE* p.C282Y homozygous (+/+) genotype is present in up to 1 in 150 persons and is the predominant cause of hemochromatosis.^1,3^ Studies of p.C282Y+/+ individuals in population samples reported low clinical penetrance, although estimates have increased with increasing sample size and inclusion of older ages. Beutler et al^6^ (n = 152 p.C282Y+/+) found that only 1% had hemochromatosis symptoms in Californian health appraisal clinics after excluding diagnosed patients. The Hemochromatosis and Iron Overload Screening Study (HEIRS) examined 36,962 men and 62,749 women (median age 50 y [range: 25–100]) and reported liver complications as the only disease associated in 299 p.C282Y+/+ males (odds ratios [OR] = 3.28, 95% CI: 1.49–7.22), with no association in female p.C282Y+/+ (OR = 0.60, 95% CI: 0.15–2.44) compared to those without p.C282Y or p.H63D *HFE* variants.^7^

In the Melbourne Collaborative Cohort Study,^8^ 28.4% (95% CI: 18.8%–40.2%) of male p.C282Y+/+ (n = 95, mean age 65 at follow-up) and 1.2% female p.C282Y+/+ had “documented iron–overload–related disease.” In UK Biobank, which includes over 2800 p.C282Y+/+ with substantial numbers now in their seventies and eighties, we found that p.C282Y+/+ is associated with excess liver fibrosis/cirrhosis,^9^ liver cancer,^9^ diabetes,^10^ osteoarthritis,^10,11^ joint replacement surgery,^11,12^ dementia,^13^ Parkinson’s disease,^12^ prostate cancer,^14^ and all-cause mortality in males.^12^ In p.C282Y+/+ females, there was excess liver fibrosis/cirrhosis, joint replacement surgery, and osteoarthritis.^12^ However, in both p.C282Y+/+ men and women, a majority would not develop these outcomes by age 80.^12^ Also, within UK Biobank, there was no statistically significant excess fatigue, morbidity, or mortality in men or women with p.C282Y/p.H63D compound homozygosity, p.H63D homozygosity, or any *HFE* heterozygous variants.^12^

Potential explanations for why only some within community genotyped p.C282Y+/+ groups develop hemochromatosis include additional smaller effect common genetic susceptibilities altering iron metabolism and endpoint disease risks,^15^ plus sex, higher alcohol consumption,^16^ other causes of liver disease, as well as adiposity. Men typically have higher iron levels than women who experience menstrual losses, and male p.C282Y+/+ more frequently experience iron overload–related health outcomes compared to p.C282Y+/+ women.^2,7^

Obesity, and especially central adiposity, is associated with similar complications to those seen in p.C282Y+/+ subjects,^12^ including type 2 diabetes (T2D),^17^ non-alcoholic fatty liver disease (NAFLD),^18^ nonalcoholic steatohepatitis,^18^ osteoarthritis,^17^ and neurodegenerative diseases.^19^ Also, obesity can increase risks of liver fibrosis/cirrhosis and hepatocellular carcinoma, likely through oxidative stress caused by NAFLD and nonalcoholic steatohepatitis.^20,21^ Powell et al^22^ reported that NAFLD was associated with increased prevalence of liver fibrosis in 214 clinically diagnosed p.C282Y+/+ patients with hemochromatosis (OR: 4.3, 95% CI: 2.1–8.8; *p* = <0.001) and that when body mass index (BMI) was substituted for steatosis in their model, BMI was independently associated with fibrosis.

Waist-to-hip ratio (WHR) provides an easily ascertained assessment of central fat distribution in routine clinical practice and is a better predictor of adverse health outcomes than BMI,^23,24^ especially for liver-related outcomes.^25^ In addition, WHR is a powerful predictor of NAFLD.^26–28^ However, evidence on the effect of larger WHR or BMI-defined obesity on penetrance to liver complications, diabetes, or arthropathy in p.C282Y+/+ persons identified in general population samples is scarce.^22,29^ We therefore aimed to estimate the effect of central adiposity, measured by WHR, and BMI-defined obesity in modifying clinical penetrance to outcomes in male and female p.C282Y+/+, using the UK Biobank community cohort.

## METHODS

### Study population

UK Biobank includes 502,464 community volunteers with baseline ages 39–73 years, recruited from 22 centers in the United Kingdom (England, Scotland, and Wales) from 2006 to 2010. Participants were moderately healthier than the general population^30^; however, *HFE* allele frequencies were comparable to other UK-based studies.^9^ Our studied sample included participants (n = 450,401) genetically similar to the 1000 genome project European Ancestry superpopulation (“EUR-like”)^31^; the categorization of this population is defined elsewhere,^32^ with *HFE* p.C282Y (rs1800562) and *HFE* p.H63D (rs1799945) genotype information from whole exome sequencing (methods by Regeneron^33^). In all, 2899 p.C282Y+/+ participants were included in the main analyses. Important health findings at baseline were reported to participants, but patient consent did not include feedback on later results, including genotypes, so study outcomes were from routine care. UK Biobank obtained ethics approval from the Northwest Multi-centre Research Ethics Committee (reference: 11/NW/0382).

### Baseline variables

Baseline variables included WHR, BMI, fibrosis-4 (FIB-4) scores, hemoglobin A1c, doctor-diagnosed conditions (including hemochromatosis, viral hepatitis, and alcoholic liver disease), and demographic and lifestyle factors (including alcohol intake frequency, smoking status, education, socioeconomic status, and physical activity). A FIB-4 score is a noninvasive estimate of liver fibrosis,^34^ calculated using platelet count and liver enzyme levels. Details on how data were recorded, calculated, and categorized can be found in the Supplemental Material, http://links.lww.com/HEP/I613.

### Incident health outcomes

Follow-up data covered hospital records from baseline to October 2022 across England, Wales, and Scotland, with cancer registries up to December 2020 for England and Wales and November 2021 for Scotland (mean follow-up: 13.3 y). Disease identification used International Classification of Diseases 10th revision (ICD-10) codes, and surgical procedures were categorized with OPCS Classification of Interventions and Procedures version 4 (OPCS-4). Prevalent diagnoses were determined using self-reported data and hospital inpatient records from 1996 to the baseline date. Incident diagnoses excluded any baseline prevalent diagnosis for the corresponding disease.

Studied incident diagnoses were selected based on our most significant previous findings, which were associated with the p.C282Y+/+ *HFE* genotype^12^; for male p.C282Y+/+, this included liver fibrosis/cirrhosis, liver cancer, T2D, osteoarthritis, joint replacement surgeries, and dementia; and for female p.C282Y+/+, this included liver fibrosis/cirrhosis, osteoarthritis, and joint replacement surgeries. Having “joint replacement surgeries” included hip, knee, ankle, or shoulder replacement surgery. A priori knowledge of central adiposity being related to NAFLD^26–28^ provided a rationale to include this additional outcome within our study (see Supplemental Table S1, http://links.lww.com/HEP/I613, for ascertainment codes for incident outcomes).

### Primary care data

Primary care records were available for a subset of p.C282Y+/+ within UK Biobank (n = 1386/2899, 47.8%), providing additional incident disease diagnoses from baseline, but records are only available until 2016–2017: that is, a mean follow-up from baseline of 7.5 years. Studied outcomes listed above were ascertained in primary care Read codes (a hierarchical clinical classification system,^35^ see Supplemental Table S1, http://links.lww.com/HEP/I613, for codes used); however, joint replacement surgeries were not analyzed as they were likely to be captured within the hospital OPCS-4 codes and not within primary care data.

### Statistical analysis

We used sex-specific spline regression plots to assess whether associations between WHR (as a continuous variable) and risk of our studied hospital-diagnosed incident outcomes within the whole UK Biobank sample (n = 450,401) and the p.C282Y+/+ genotype group (n = 2899) were linear. We used Cox proportional hazards regression models using the continuous WHR variable as the exposure variable to determine its association with the risk of incident outcomes in p.C282Y+/+ (sex-stratified) while adjusting for age and 10 genetic principal components; adjusting for principal components reduces bias due to population stratification.^36^ In addition, effect estimates were reported in units of standard deviation (SD) from the mean. We also used established sex-specific WHR cutoffs (high WHR ≥0.96 for men and ≥0.85 for women) and BMI cutoffs as underweight (<18.5 kg/m^2^), normal (reference group: 18.5–24.9 kg/m^2^), overweight (25–29.9 kg/m^2^), and obese (≥30 kg/m^2^), which have previously been shown to be indicators of central adiposity or obesity respectively, and are associated with adverse health outcomes.^37–39^ Cox proportional hazards regression models were used to test the association between this binary WHR variable and BMI groups in separate models to test the risk of incident outcomes by the end of follow-up, in p.C282Y+/+ (n = 2899). Models were stratified by sex and adjusted for age and 10 genetic principal components^36^ (model 1). Kaplan-Meier survivor functions estimated cumulative incidence of associated outcomes within p.C282Y+/+, stratified by WHR status from age 40 to 80 years. We calculated the population attributable fraction (PAF) to measure how much of our observed health outcomes were attributable to a high WHR within p.C282Y+/+, using the “punafcc” post-estimation command after Cox regression in Stata.^40^

We tested for an interaction between p.C282Y+/+ genotype and the binary WHR variable and the categorized BMI variable separately on the risk of outcomes compared to those with no *HFE* p.C282Y or p.H63D genotypes, n = 268,007); this analysis was used to test for a multiplicate effect of the p.C282Y+/+ genotype and a high WHR/BMI groups on our studied outcomes.

All analyses were conducted using the statistical software Stata version 18.0, except the spline point regression analyses, which used R v4.3.1 and package “pspline” v1.0-19.

### Sensitivity analysis

In sensitivity analysis, we further adjusted Cox regression models for potential confounders of central adiposity/obesity and outcomes, which included alcohol intake, smoking status, education, Townsend deprivation index, physical activity, and any baseline diagnosis of viral hepatitis or alcoholic liver disease (model 2).

We also repeated Cox regression analyses within p.C282Y+/+ participants, excluding those with diagnosed hemochromatosis at baseline (n = 1140 males and n = 1548 females); a diagnosis of hemochromatosis typically indicates an individual undergoing iron monitoring and treatment required to improve/prevent the progression of the disease. Cox regression analyses were also repeated in a subset of p.C282Y+/+ participants with additional primary care data available (n = 613 males and n = 773 females).

## RESULTS

We studied 2899 p.C282Y+/+ participants (mean follow-up: 13.3 years; 1297 males and 1602 females). The mean age of males and females at baseline assessment was 56.8 (SD: 8.2) and 56.9 (SD: 8.0) years, respectively. Our sample included 440 (33.9%) male p.C282Y+/+ with a high WHR and 480 (30.0%) female p.C282Y+/+, of which 70 (15.9%) and 18 (3.8%) were diagnosed with hemochromatosis at baseline, respectively. Baseline diagnoses of NALFD in male and female p.C282Y+/+ were low (n <5; n<5), respectively (Table 1 and Supplemental Table S2, http://links.lww.com/HEP/I613, for comparison of baseline characteristics of participants without *HFE* p.C282Y or p.H63D genotypes). At baseline, hemoglobin A1c and WHR showed a positive linear relationship within male p.C282Y+/+ (β = 3.20, 95% CI: 2.30–4.09, *p* = 3.5 × 10^−12^); a similar effect was observed in males without *HFE* variants (β = 2.92, 95% CI: 2.84–3.01, *p* = 0.0 × 10^−<99^). In female C282Y+/+ and females without *HFE* variants, we observed a similar relationship with hemoglobin A1c and WHR (β = 1.57, 95% CI: 0.99–2.14, *p* = 9.4 × 10^−8^ and β = 2.24, 95% CI: 2.18–2.30, *p* = 0.0 × 10^−<99^), respectively.

**TABLE 1:** Baseline characteristics of male and female p.C282Y+/+ within UK Biobank

	Male p.C282Y+/+	Females p.C282Y+/+
Total participants, n	1297	1602
Participants with primary care records, n	613	773
Diagnosed hemochromatosis, n (%)	157 (12.1)	54 (3.4)
Mean age, y (SD)	56.84 (8.2)	56.92 (8.0)
WHR (SD)	0.94 (0.1)	0.82 (0.1)
WHR ≥0.96, n (%)	440 (33.92)	—
WHR ≥0.85, n (%)	—	480 (29.96)
BMI, kg/m^2^ (SD)	27.63 (4.1)	26.92 (5.1)
Underweight (<18.5 kg/m^2^), n (%)	5 (0.39)	13 (0.81)
Normal (18.5–24.9 kg/m^2^), n (%)	335 (25.87)	647 (40.46)
Overweight (25–29.9 kg/m^2^), n (%)	662 (51.12)	589 (36.84)
Obese (≥30 kg/m^2^), n (%)	293 (22.63)	350 (21.89)
FIB-4 score, n (%)		
Advanced fibrosis likely (>2.67)	56 (4.67)	37 (2.51)
NAFLD, n (%)	<5	<5
HbA1c (mmol/mol) (SD)	39.92 (7.77)	33.66 (5.42)
Viral hepatitis, n (%)	7 (0.54)	7 (0.44)
Alcoholic liver disease, n (%)	6 (0.46)	0 (0.00)
Alcohol intake frequency, n (%)		
Never	82 (6.32)	132 (8.24)
Low	192 (14.80)	440 (27.47)
Moderate	355 (27.37)	441 (27.55)
High	668 (51.50)	589 (42.53)
Ever smoked, n (%)	694 (53.72)	681 (42.51)
Highest educational qualification, n (%)		
None	264 (20.59)	283 (17.85)
CSEs	48 (3.74)	61 (3.85)
GCSEs/O-levels	138 (10.76)	231 (14.57)
A-levels/NVQ/HND/HNC	262 (20.44)	267 (16.85)
Professional qualification	173 (13.49)	289 (18.23)
University degree	397 (30.97)	454 (28.64)
Top quartile of Townsend index, n (%)	350 (27.01)	429 (26.76)
Top quartile of MET minutes per week, n (%)	295 (26.84)	276 (21.87)

*Note*: Male and female p.C282Y homozygous participants (n = 2899) genetically similar to the 1000 genome project European Ancestry superpopulation (“EUR-like”) with *HFE* genotypic data available in the UK Biobank. The numbers presented are mean (SD) for continuous variables and n (%) for categorical variables. Cutoffs for WHR were ≥0.96 for men and ≥0.85 for women. See Supplemental Table S2, http://links.lww.com/HEP/I613, for baseline characteristics of male and females without *HFE* p.C282Y or p.H63D genotypes.

Abbreviations: A-level, advanced level; BMI, body mass index; CSEs, Certificate of Secondary Education; FIB-4, fibrosis-4; GCSEs, General Certificate of Secondary Education; HbA1c, hemoglobin A1c; HNC, Higher National Certificate; HND, Higher National Diploma; MET, metabolic equivalent task; NVQ, National Vocational Qualification; O-level, ordinary level; SD, standard deviation; WHR, waist-hip-ratio; +/+, homozygous.

To validate previously reported clinical cutoffs for central adiposity^37–39^ in UK Biobank, we estimated the association between WHR and the primary outcomes studied (liver fibrosis and cirrhosis, liver cancer, NAFLD, and T2D). We used Cox proportional hazards regression models in all p.C282Y/p.H63D genotype groups (n = 450,401), stratified by sex, and included a spline term for WHR; we found that WHRs ≥0.96 for men and ≥0.85 for women were appropriate cutoffs to identify participants with increased risk of our studied outcomes (Supplemental Figures S1A–D and S2A, B, http://links.lww.com/HEP/I613).

### Male p.C282Y+/+ and risk of outcomes

**FIGURE 1:**
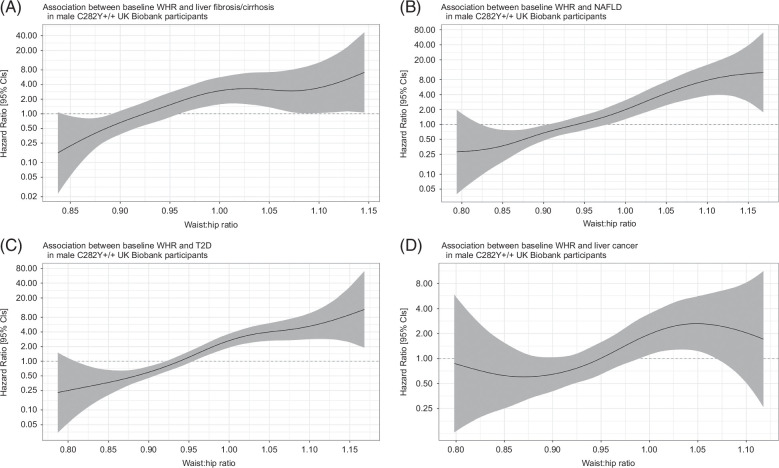
Spline regression for the association between WHR and risk of incident outcomes in male p.C282Y+/+ (n=1,297). Each model was adjusted for age and principal components 1-10. Abbreviations:, +/+, homozygous; CI, confidence interval; NAFLD, non-alcoholic fatty liver disease; WHR, waist-to-hip ratio.

Within male p.C282Y+/+, spline regression plots showed a positive linear association between WHR and risk of incident disease (Figures 1A–D). WHR as a continuous variable (in units of SD from the mean) increased risk of liver fibrosis/cirrhosis (n = 36, hazard ratio [HR] per SD: 1.95, 95% CI: 1.43–2.66, *p* = 2.4 × 10^−5^), liver cancer (n = 31, HR per SD: 1.63, 95% CI: 1.14–2.32, *p* = 0.007), NAFLD (n = 57, HR per SD: 2.27, 95% CI: 1.77–2.90, *p* = 7.7 × 10^−11^), and T2D (n = 115, HR per SD: 2.15, 95% CI: 1.78–2.58, *p* = 5.1 × 10^−16^).

**FIGURE 2:**
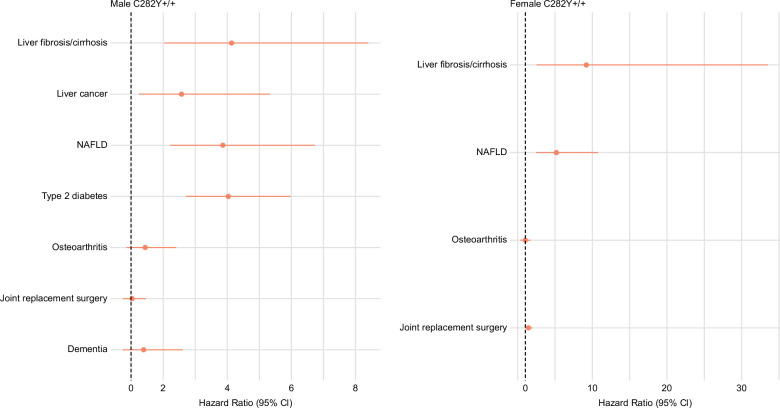
Hazard ratios for incident hospital diagnosed outcomes in male and female p.C282Y+/+ with high WHR compared to normal WHR, adjusted for age and principal components 1-10. Outcomes were analysed based on their association with p.C282Y+/+ in our previous research12. See supplementary eTables 3 & 6 for incident numbers and HRs. Abbreviations: +/+, homozygous; CI, confidence interval; NAFLD, non-alcoholic fatty liver disease; WHR, waist-to-hip ratio.

**FIGURE 3:**
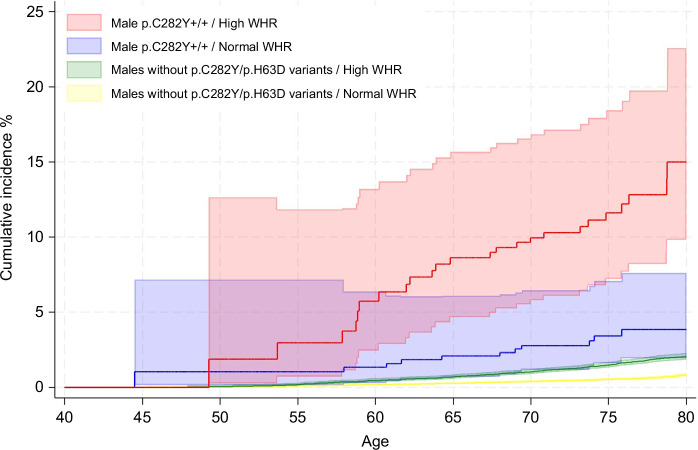
Kaplan-Meier curves for the cumulative incidence of liver fibrosis or cirrhosis in male p.C282Y+/+ and those without HFE p.C282Y or p.H63D variants to age 80 by WHR status. Cumulative incidence (estimated % diagnosed by age 80 years; 95% CIs), excluding baseline cases of fibrosis/cirrhosis. Abbreviations: +/+, homozygous; WHR, waist-to-hip ratio.

In p.C282Y+/+ males, having a high WHR was associated with increased odds of advanced fibrosis based on a high FIB-4 score at baseline (OR = 1.91, 95% CI: 1.10–3.31, *p* = 0.02). Hospital inpatient–diagnosed liver fibrosis/cirrhosis incidence increased in male p.C282Y+/+ with a high WHR (HR = 4.13, 95% CI: 2.04–8.39, *p* = 8.4 × 10^−5^) with a cumulative incidence by age 80 of 15.0% (95% CI: 9.8%–22.6%) versus 3.9% (95% CI: 1.9%–7.6%) compared to those with a normal WHR (Figures 2, 3 and Supplemental Tables S3 and S4, http://links.lww.com/HEP/I613). By the end of follow-up, hemochromatosis had also been diagnosed in 23 (96%) of the 24 high WHR male p.C282Y+/+ participants who developed liver fibrosis/cirrhosis (Supplemental Table S3, http://links.lww.com/HEP/I613). For comparison, in study subjects without p.C282Y or p.H63D variants, the cumulative incidence of liver fibrosis/cirrhosis by age 80 (Figure 3 and Supplemental Table S4, http://links.lww.com/HEP/I613) with high WHR was 2.0% (95% CI: 1.8%–2.3%) and with normal WHR was 0.8% (95% CI: 0.7%–1.0%). In male p.C282Y+/+, half of all liver fibrosis/cirrhosis cases within the population were statistically attributable to having a high WHR (PAF = 50.5%, 95% CI: 37.7%–60.7%). Male p.C282Y+/+ with a high WHR also had an increased risk of liver cancer (HR = 2.57, 95% CI: 1.24–5.33, *p* = 0.01; PAF = 37.4%, 95% CI: 17.3%–52.6%) (Figure 2 and Supplemental Table S3, http://links.lww.com/HEP/I613). By age 80, the cumulative incidence of liver cancer in male p.C282Y+/+ was 9.2% (95% CI: 5.7%–14.6%) in those with a high WHR versus 3.6% (95% CI: 1.9%–6.6%) in those with a normal WHR (Supplemental Table S4, http://links.lww.com/HEP/I613).

Male p.C282Y+/+ with high WHR had greater risk of NAFLD (HR = 3.86, 95% CI: 2.22–6.72, *p* = 1.8 × 10^−6^; PAF = 45.5%, 95% CI: 35.9%–53.6%) compared to those with normal WHR (Figure 2 and Supplemental Table S3, http://links.lww.com/HEP/I613). Cumulative incidence by age 80 of NAFLD in high WHR p.C282Y+/+ males was 20.9% (95% CI: 14.8%–29.1%) versus 6.4% (95% CI: 4.2%–9.7%) in normal WHR (Supplemental Table S4, http://links.lww.com/HEP/I613).

Male p.C282Y+/+ with a high WHR also had an increased risk of T2D (HR = 4.03, 95% CI: 2.71–5.98, *p* = 5.3 × 10^−12^; PAF = 50.3%, 95% CI: 43.3%–56.5%) with a significantly greater cumulative incidence of T2D diagnosed by age 80 compared to those with normal WHR (45.0%, 95% CI: 35.8%–55.3% vs. 11.8%, 95% CI: 8.4%–16.4%) (Figure 2 and Supplemental Tables S3 and S4, http://links.lww.com/HEP/I613).

We found no statistical association between osteoarthritis, joint replacement surgeries, or dementia in male p.C282Y+/+ with a high WHR compared to those with a normal WHR (Figure 2 and Supplemental Table S3, http://links.lww.com/HEP/I613).

Using BMI as an alternative exposure variable, obese (≥30 kg/m²) male p.C282Y+/+ were not at a significantly increased risk of liver fibrosis/cirrhosis or liver cancer compared to those with normal BMI (Supplemental Table S5, http://links.lww.com/HEP/I613). However, they had an increased risk of NAFLD (HR = 4.52, 95% CI: 2.05–9.97, *p* = 0.0002) and T2D (HR = 9.37, 95% CI: 4.46–19.69, *p* = 3.6 × 10^−9^) compared to those with a normal BMI. Male p.C282Y+/+ with obesity had an increased risk of joint replacement surgery (HR = 1.81, 95% CI: 1.09–2.99, *p* = 0.02), in contrast to those with high WHR (HR = 1.03, 95% CI: 0.74–1.45, *p* = 0.85); however, this risk was not statistically significant in model 2 (Supplemental Table S5, http://links.lww.com/HEP/I613).

### Female p.C282Y+/+ and risk of outcomes

**FIGURE 4:**
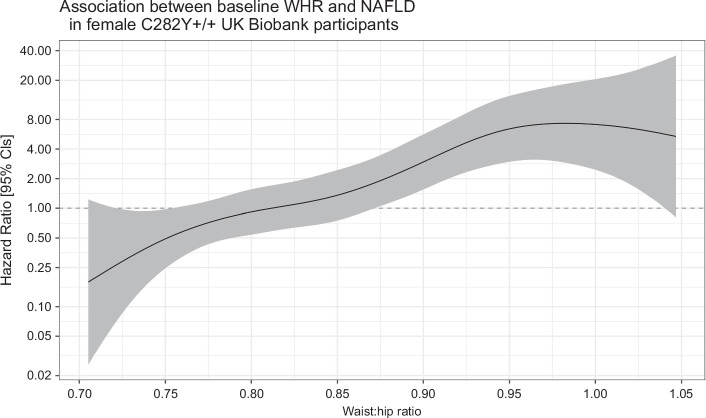
Spline regression for the association between WHR and risk of NAFLD incidence in female p.C282Y+/+ (n=1,602). The model was adjusted for age and principal components 1-10. Abbreviations: +/+, homozygous; CI, confidence interval; NAFLD, non-alcoholic fatty liver disease; WHR, waist-to-hip ratio.

**FIGURE 5:**
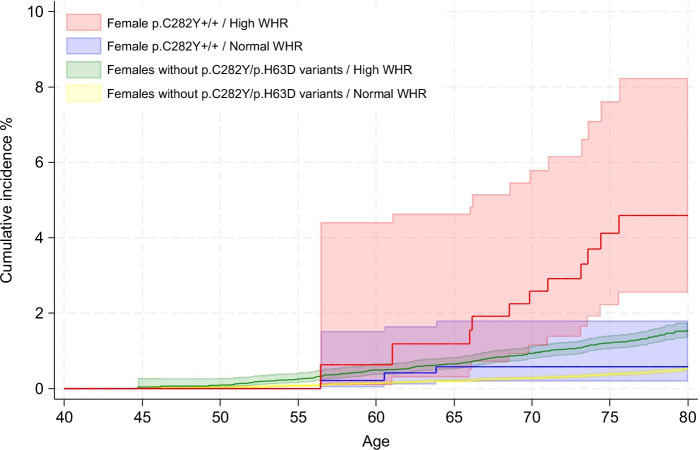
Kaplan-Meier curves for the cumulative incidence of liver fibrosis or cirrhosis in female p.C282Y+/+ and those without HFE p.C282Y or p.H63D variants to age 80 by WHR status. Cumulative incidence (estimated % diagnosed by age 80 years; 95% CIs), excluding baseline cases of fibrosis/cirrhosis. Abbreviations: +/+, homozygous; WHR, waist-to-hip ratio.

Spline regression showed a linear association in female p.C282Y+/+ between WHR and risk of NAFLD (n = 33, HR per SD: 2.37, 95% CI: 1.73–3.24, *p* = 6.9 × 10^−8^; Figure 4). A similar association was observed for liver fibrosis/cirrhosis risk (n = 14, HR per SD: 2.51, 95% CI: 1.57–4.02, *p* = 1.2 × 10^−4^); however, sample numbers were too small to produce spline regression plots to visualize this effect. At baseline, having a high WHR was trending, but not statistically significant, toward an increased odds of advanced fibrosis based on a high FIB-4 score at baseline (OR = 1.73, 95% CI: 0.89–3.38, *p* = 0.11). Hospital inpatient–diagnosed liver fibrosis/cirrhosis incidence was significantly increased in high WHR p.C282Y+/+ females (HR = 9.17, 95% CI: 2.51–33.50 *p* = 8.0 × 10^−4^; PAF = 70.0%, 95% CI: 56.6%–79.3%), who had a greater proportion of diagnoses by age 80 (4.6%, 95% CI: 2.5%–8.2% vs. 0.6%, 95% CI: 0.2%–1.8%) (Figures 2 and 5 and Supplemental Tables S4 and S6, http://links.lww.com/HEP/I613) compared with normal WHR. By the end of follow-up, hemochromatosis had also been diagnosed in 9 (82%) of the 11 high WHR female p.C282Y+/+ participants who developed liver fibrosis/cirrhosis (Supplemental Table S6, http://links.lww.com/HEP/I613).

High WHR p.C282Y+/+ females had an increased risk of NAFLD (HR = 5.17, 95% CI: 2.48–10.78, *p* = 1.2 × 10^−5^; PAF = 51.3%, 95% CI: 41.4%–59.6%). The cumulative incidence of NAFLD was 8.9% (95% CI: 5.7%–13.7%) versus 2.7% (95% CI: 1.4%–5.3%) (Figure 2 and Supplemental Tables S4 and S6, http://links.lww.com/HEP/I613). Female p.C282Y+/+ with a high WHR also had an increased risk of joint replacement surgeries (HR = 1.42, 95% CI: 1.03–1.96, *p* = 0.03; PAF = 11.8%, 95% CI: 2.2%–20.4%) (Figure 2 and Supplemental Table S6, http://links.lww.com/HEP/I613). No association was found between a high WHR and the incidence of hospital-diagnosed osteoarthritis in female p.C282Y+/+ by the end of the follow-up.

In female p.C282Y+/+ with a BMI ≥30 kg/m², the risk of liver fibrosis/cirrhosis was not significantly increased (HR = 3.35, 95% CI: 0.78–14.35, *p* = 0.10). The risk of NAFLD was comparable to the effect estimate observed using WHR (HR = 5.20, 95% CI: 1.84–14.69, *p* = 0.002) (Supplemental Tables S6 and S7, http://links.lww.com/HEP/I613, for comparison). Female p.C282Y+/+ who were overweight (25–29.9 kg/m^2^) also had an increased risk of NAFLD (HR = 3.34, 95% CI: 1.21–9.21, *p* = 0.02) compared to normal BMI. Both overweight and obese female p.C282Y+/+ had a greater risk of a joint replacement surgery (HR = 1.59, 95% CI: 1.08–2.33, *p* = 0.02 and HR = 1.92, 95% CI: 1.27–2.90, *p* = 0.002, respectively) (Supplemental Table S7, http://links.lww.com/HEP/I613).

### Interaction analysis

We tested for interactions between the p.C282Y+/+ genotype and binary WHR and BMI categories separately on the risk of outcomes compared to those with no *HFE* p.C282Y or p.H63D genotypes. We found no significant interactions between high WHR and p.C282Y+/+ genotype in males or females, respectively, for any incident diagnoses (Supplemental Tables S8 and S9, http://links.lww.com/HEP/I613). There was a significant interaction between the obese BMI group and male p.C282Y+/+ for osteoarthritis (*p* = 0.01); however, no statistical interactions were found between BMI groups and other incident diagnoses for male or female p.C282Y+/+ (Supplemental Tables S10 and S11, http://links.lww.com/HEP/I613).

### Sensitivity analysis

In models further adjusted for alcohol intake, education, smoking status, Townsend deprivation index, and any baseline diagnosis of viral hepatitis or alcoholic liver disease, the risk of outcomes remained similar in male and female p.C282Y+/+ with a high WHR. However, the risk of joint replacement surgeries in female p.C282Y+/+ became nonsignificant (Supplemental Tables S3 and S6, http://links.lww.com/HEP/I613, model 2). After adjustment, effect estimates for T2D risk were slightly increased in overweight and obese male p.C282Y+/+ and their risk of joint replacement surgery became nonsignificant in the obese BMI group (Supplemental Table S5, http://links.lww.com/HEP/I613). After excluding baseline cases of hemochromatosis in p.C282Y+/+ males (n = 157) and females (n = 54), associations between a high WHR and outcomes remained consistent (Supplemental Tables S12 and S13, http://links.lww.com/HEP/I613).

In the subset of p.C282Y+/+ participants with additional primary care health records, associations between having a high WHR and an increased risk of liver fibrosis or cirrhosis, compared to a normal WHR, were similar to using only hospital inpatient diagnoses; males (n = 613, HR = 5.73, 95% CI: 2.06–15.92, *p* = 8.3×10^−4^) and females (n = 773, HR = 10.70, 95% CI: 1.96–58.47, *p* = 6.2 × 10^−3^) (Supplemental Tables S14 and S15, http://links.lww.com/HEP/I613). In male p.C282Y+/+ with a high WHR, hazards for liver cancer became nonsignificant (HR = 1.89, 95% CI: 0.69–5.16, *p* = 0.21), likely due to the reduced sample size.

## DISCUSSION

The hemochromatosis-associated *HFE* p.C282Y+/+ genotype and central adiposity are separately associated with similar health-related outcomes, including liver complications, T2D, and arthropathy. While there is some evidence of the importance of adiposity in p.C282Y+/+ clinical patients, effects in community-identified p.C282Y+/+ were unclear. We found that within p.C282Y+/+ males, central adiposity strongly predicted liver fibrosis/cirrhosis, liver cancer, NAFLD, and T2D, and liver fibrosis/cirrhosis and NAFLD in females. The combined effects of p.C282Y+/+ and high WHR on cumulative incidence by age 80 in males were substantial; for example, for liver fibrosis/cirrhosis risk, male p.C282Y+/+ with a high WHR cumulative incidence by age 80 was 15.0% (95% CI: 9.8%–22.6%) versus 3.9% (95% CI: 1.9%–7.6%) with a normal WHR (Figures 2 and 3 and Supplemental Tables S3 and S4, http://links.lww.com/HEP/I613); however, in males without p.C282Y or p.H63D variants, the respective estimates (Figure 3 and Supplemental Table S4, http://links.lww.com/HEP/I613) were only 2.0% (95% CI: 1.8%–2.3%) and 0.8% (95% CI: 0.7%–1.0%). The great majority of both male (23 of 24) and female (9 of 11) p.C282Y+/+ high WHR participants who developed liver fibrosis/cirrhosis were also diagnosed with hemochromatosis by the end of follow-up.

### Comparison to previous obesity and iron studies

Direct comparison of findings with previous studies is difficult, given the scarcity of data from other community genotyped cohorts. Powell et al^22^ reported that steatosis was associated with increased prevalence of liver fibrosis in 214 clinically diagnosed hemochromatosis patients with p.C282Y+/+ (OR = 4.3, 95% CI: 2.1–8.8; *p* = <0.001). Our sample of baseline-diagnosed NAFLD cases was too small to replicate Powell et al’s analyses directly. Instead, we have analyzed associations with greater central adiposity, measured by WHR, a powerful risk factor for NAFLD.^26–28^ A previous report that used 216 single nucleotide polymorphisms to create a polygenic risk score for high WHR, while adjusting for BMI, reported an association with NAFLD (OR = 1.42, 95% CI: 1.24–1.62, *p* = 4.7 × 10^−7^) and liver fat accumulation, which was similar when replicated in separate cohorts.^28^ Other observational studies have reported similar associations whereby WHR showed a strong positive linear relationship for an increase in fatty liver index score (β = 27.48; 95% CI: 23.48–31.48),^26^ which is a validated tool to measure hepatic steatosis^41^ with an accuracy of (AUC) 0.84 (95% CI: 0.81–0.87).^42^ Moreover, Zheng et al^27^ reported that WHR is the most accurate anthropometric measurement for identifying NAFLD with an AUC of 0.92 (95% CI: 0.86–0.97, *p* = 2.6 × 10^−3^) compared to BMI (0.85) and WC (0.88). Therefore, WHR should be considered as a reliable anthropometric measurement to predict NAFLD, even more so within p.C282Y+/+, as our findings show that both males and females had significantly greater risk of the disease with a high WHR, even at older ages.

Powell et al^22^ also found that within patients with hemochromatosis with p.C282Y+/+, BMI was independently associated with liver fibrosis in both light alcohol drinkers (OR = 2.2, 95% CI: 1.2–4.2; *p* = 0.013) and moderate-heavy alcohol drinkers (OR = 3.6, 95% CI: 1.2–10.6; *p* = 0.023). Although we found a significant association between central adiposity (high WHR) and an increased risk of liver fibrosis/cirrhosis in both male and female UK Biobank participants, we found no association between BMI-defined overweight or obesity and risk of liver fibrosis/cirrhosis. Barton et al^29^ reported that a BMI ≥30.0 kg/m^2^ was no more prevalent among p.C282Y+/+ (n = 66) with NAFLD compared to those without NAFLD. However, their samples of obese p.C282Y+/+ with and without NAFLD were modest for comparison purposes (n = 3 and n = 8, respectively). Nonetheless, despite this nonsignificant difference, our results show that male and female p.C282Y+/+ with an obese BMI were at a greater risk of being diagnosed with NAFLD (HR = 4.52, 95% CI: 2.05–9.97, *p* = 0.0002 and HR = 5.20, 95% CI: 1.84–14.69, *p* = 0.002), respectively, when compared to those with a normal BMI.

Although weight loss is regarded as a clinical characteristic in *HFE* patients with hemochromatosis,^43,44^ we found no difference in high WHR prevalence comparing p.C282Y+/+ males or females with those without *HFE* p.C282Y or p.H63D variants (*p* = 0.89 and *p* = 0.65, respectively). We similarly found no differences in BMI between male p.C282Y+/+ and males without *HFE* variants (27.63 vs. 27.85 kg/m^2^; *p* = 0.06), which was the same for females (26.92 vs. 27.02 kg/m^2^; *p* = 0.46). Previous reports of associations between BMI and p.C282Y+/+ in the general population have varied: a study of 67 p.C282Y+/+ males over the age of 40 years showed significantly lower BMIs compared to their male siblings without the same *HFE* mutation (n = 15) and also a comparable National Health and Nutrition Examination Survey male cohort (26.7 vs. 30.5 kg/m^2^ and 28.7 kg/m^2^, *p* < 0.05),^45^ respectively; the difference in females was nonsignificant.^45^ Similarly, there was no difference in BMI from a US atherosclerosis risk screening program between p.C282Y+/+ (n = 44) and those without *HFE* variants (n = 6768) (26.5 vs. 26.9 kg/m^2^), respectively.^46^

In apparent contrast to the Powell et al^22^ study and our findings, Laine et al^47^ studied 57 female and 21 male p.C282Y homozygotes from a French community screening program (mean age approximately 45) and found that increasing BMI was associated with lower rates of clinical expression of hemochromatosis (based on transferrin saturation) in the women (in models adjusting for various factors including ferritin levels). Deugnier et al^48^ studied 2050 p.C282Y+/+ patients diagnosed with hemochromatosis over a 30-year period in France, and found time trends of reducing iron levels and reducing liver complications, which modeling attributed in part to decreasing alcohol intakes and increasing obesity. In the general population, there is evidence that obesity is associated with a decrease in iron status.^49^ However, an MRI-based study in a subset of the UK Biobank participants (of all genotypes) reported genetic evidence for higher liver iron levels with increasing central adiposity.^50^ Unfortunately, the numbers for p.C282Y+/+ and liver MRI in UK Biobank are currently too small to explore this issue directly. More work is clearly needed on the likely complex interacting biological effects involved in the combination of p.C282Y+/+ and adiposity. Such studies should clarify whether the divergence of findings on the effects of adiposity is due to our inclusion of incident outcomes to older ages (thereby reducing volunteer study responder biases and reverse causation) and including well-powered samples of men.

### Strengths and limitations

We studied a large community-based cohort using prospectively collected diagnosis records, providing the first large-scale analysis of central adiposity, defined as a high WHR and incident disease in p.C282Y+/+ groups. WHR is a reliable measure of central fat distribution and increased risk of health-related outcomes when compared to other measures such as BMI.^23,24^ Allele frequencies of the *HFE* gene were comparable to previous UK studies.^9^ We studied incident outcomes during a mean 13.3-year follow-up to reduce any healthy volunteer bias at recruitment to UK Biobank at baseline.^30^ Cox proportional hazard models met statistical assumptions. Our main analyses are based on outcomes recorded mainly in hospital inpatient care,^9^ but similar results were obtained in the smaller and shorter follow-up primary care records subset in males and females for the more common liver outcomes (liver fibrosis and cirrhosis). Our findings of raised liver cancer risks in male p.C282Y+/+ with high WHR (including cancer registry data covering outpatient and inpatient diagnoses) provide a robust endpoint for the study. Incident disease samples for specific outcomes were limited due to stratification by sex and grouping by WHR/BMI status; thus, some effect estimates may have been underpowered.

Liver complications are most common in p.C282Y+/+ individuals with substantially raised transferrin saturation or serum ferritin measures,^1^ but unfortunately, UK Biobank lacks these blood iron data. It is, therefore, possible that the influence of WHR on the studied health outcomes could be due to non-iron mechanisms in p.C282Y+/+ participants with no biological expression (ie, with normal iron measures and no hemochromatosis). However, the electronic health records studied reveal that the great majority of both male (23 of 24) and female (9 of 11) p.C282Y+/+ high WHR participants who developed liver fibrosis/cirrhosis were also clinically diagnosed with hemochromatosis by the end of follow-up, indicating that treating physicians considered iron-related disease to be present. Further work is needed on the relative contributions of excess iron and other factors to liver and diabetes complications in p.C282Y+/+ individuals. This work will be challenging, as raised ferritin is also common with central adiposity and related metabolic syndrome features^51^; thus, intervention trials to reduce adiposity may be needed, as observational studies will likely be prone to confounding. Lastly, further work is needed to establish whether the WHR (but not BMI) effects on liver fibrosis/cirrhosis incidence are due to steatotic liver disease being the main risk factor.

### Clinical implications

Heavier alcohol intakes are a recognized cofactor in hemochromatosis-related liver complications,^16,52^ and patients are therefore advised to avoid heavy drinking.^44^ However, we have found little mention of the risks of obesity or central adiposity in published advice to patients. Instead, such advice often focuses on alcohol intake, food groups, and vitamin supplements.^53,54^ Weight management is seldom recommended even in clinical guidelines^44^ despite obesity being associated with liver fibrosis/cirrhosis risk through NAFLD and Nonalcoholic Steatohepatitis.^18^ As WHR is easily measured in routine clinical practice, our results suggest this should be measured routinely to inform clinical decision-making on management and prognosis. Clinical trials are needed for active weight loss interventions to improve outcomes in p.C282Y+/+ men and women. As avoiding central adiposity is already part of health advice to the general population, adding the need to avoid high WHRs to the advice to p.C282Y+/+ subjects seems a logical step.

## CONCLUSIONS

Central adiposity, measured by a higher WHR, is a strong predictor of incident liver disease and diabetes in p.C282Y+/+ persons in the UK Biobank community genotyping. General health advice to p.C282Y homozygous persons should include the need to prevent central adiposity. Clinical trials are needed to test the effects of active weight loss interventions in *HFE* p.C282Y+/+ patients with high WHRs to improve associated clinical outcomes.

## Supplementary Material
