# Supplementary material for: Incidence of liver complications with hemochromatosis-associated HFE p.C282Y homozygosity: The role of central adiposity
Source: Hepatology. 2024 Aug 23;81(5):1522–34. doi: 10.1097/HEP.0000000000001056 (PMC11999091; doi:10.1097/HEP.0000000000001056)
Supplement: Supplementary file 1 [file hep-81-1522-s001.docx]

**Supplementary Material**

**Contents**

[**Baseline variable** 2](#_Toc171686816)

[**eTable 1.** Incident hospital diagnosed outcomes or procedures with associated ICD-10/OPCS codes and incident primary care outcomes with associated READ codes 3](#_Toc171686817)

[**eTable 2.** Baseline characteristics of male and female p.C282Y homozygotes and those without *HFE* p.C282Y or p.H63D genotypes within UK Biobank 6](#_Toc171686818)

[**eTable 3**. Risk of incident hospital diagnoses in male p.C282Y homozygous UK Biobank participants by WHR 8](#_Toc171686819)

[**eTable 4.** Cumulative incidence of outcomes from ages 40-80 years by WHR status in p.C282Y homozygous male and females and those without p.C282Y or p.H63D genotypes variants 9](#_Toc171686820)

[**eTable 5.** Risk of incident hospital diagnoses in male p.C282Y homozygous UK Biobank participants by BMI status 10](#_Toc171686821)

[**eTable 6:** Risk of incident hospital diagnoses in female p.C282Y homozygous UK Biobank participants by WHR 12](#_Toc171686822)

[**eTable 7.** Risk of incident hospital diagnoses in female p.C282Y homozygous UK Biobank participants by BMI status 13](#_Toc171686823)

[**eTable 8.** Interaction analysis for multiplicative effect of *HFE* p.C282Y homozygous males and high WHR on risk of incident hospital diagnoses compared to males without *HFE* p.C282Y or p.H63D genotypes and normal WHR 14](#_Toc171686824)

[**eTable 9.** Interaction analysis for multiplicative effect of *HFE* p.C282Y homozygous females and high WHR on risk of incident hospital diagnoses compared to females without *HFE* p.C282Y or p.H63D genotypes and normal WHR 15](#_Toc171686825)

[**eTable 10.** Interaction analysis for multiplicative effect of *HFE* p.C282Y homozygous males and BMI groups on risk of incident hospital diagnoses compared to males without *HFE* p.C282Y or p.H63D genotypes and normal BMI 16](#_Toc171686826)

[**eTable 11.** Interaction analysis for multiplicative effect of *HFE* p.C282Y homozygous females and BMI groups on risk of incident hospital diagnoses compared to females without *HFE* p.C282Y or p.H63D genotypes and normal BMI 17](#_Toc171686827)

[**eTable 12.** Risk of incident hospital diagnoses in male p.C282Y homozygous UK Biobank participants by WHR, excluding those with a diagnosis of hemochromatosis at baseline 18](#_Toc171686828)

[**eTable 13.** Risk of incident hospital diagnoses in female p.C282Y homozygous UK Biobank participants by WHR, excluding those with a diagnosis of hemochromatosis at baseline 19](#_Toc171686829)

[**eTable 14**. Risk of outcomes using incident primary care and hospital diagnoses in male p.C282Y homozygous UK Biobank participants by WHR 20](#_Toc171686830)

[**eTable 15**. Risk of outcomes using incident primary care and hospital diagnoses in female p.C282Y homozygous UK Biobank participants by WHR 21](#_Toc171686831)

[**FIGURES** 22](#_Toc171686832)

[**eFigure 1.** Spline regression for the association between WHR and risk of incident outcomes in all male UK Biobank participants 22](#_Toc171686833)

[**eFigure 2.** Spline regression for the association between WHR and risk of incident outcomes in all female UK Biobank participants 24](#_Toc171686834)

## **Baseline variable**

Weight, height, waist circumference (WC), and hip circumference (HC) were measured during initial assessment visits. We calculated BMI as weight (kg) divided by height (m^2^), and WHR by dividing WC (cm) by HC (cm). Baseline liver enzyme concentrations were measured using International Federation of Clinical Chemistry standards^1^. Platelet counts were measured using four Beckman Coulter LH750 instruments^2^. We calculated Fibrosis-4 (FIB-4) scores, a verified non-invasive estimate of liver fibrosis^3^, using the following calculation: (age x aspartate aminotransferase) ÷ (platelet count x √alanine aminotransferase). FIB-4 >2.67 was used to categorise participants at risk of advanced liver fibrosis. Baseline measures of blood glucose were measured using the hemoglobin A1c (HbA1c) test measured by high-performance liquid chromatography analysis on a Bio-Rad VARIANT II Turbo^4^.Baseline questionnaires collected information on doctor-diagnosed conditions (including hemochromatosis, viral hepatitis, and alcoholic liver disease) and demographic and lifestyle factors (including alcohol intake frequency, smoking status, education, socioeconomic status, and physical activity). Alcohol intake frequency was categorized into 4 groups: ‘Never’, ‘Low’ which consisted of those who drank one to three times per month, and only on special occasions, ‘Moderate’ which consisted of those who drank once or twice per week and ‘High’ which included those who drank three to four times per week, and daily or almost daily. Smoking status was categorized into those who had ‘Never’ previously smoked, and those who ‘Ever smoked’ which consisted of being a past or current smoker. Highest educational qualification was ranked as: 0=none, 1=CSEs (Certificate of Secondary Education), 2=GCSEs/O-levels (General Certificate of Secondary Education to age 16), 3=A-levels/NVQ/HND/HNC (further education after age 16), 4=professional qualification, and 5=college/university degree. Socioeconomic status was recorded using the Townsend deprivation index, which integrates unemployment, lack of car ownership, absence of homeownership, and household overcrowding within a participant’s region of residence^5^. A greater Townsend deprivation index indicates an increased material disadvantage. The participants' frequency and duration of walking, moderate, and vigorous exercise were collected via adapted questions from the validated International Physical Activity Questionnaire^6^. The total Metabolic Equivalent Task (MET) score was calculated based on the minutes participants exercised per week, as described previously^7^.

## **eTable 1.** Incident hospital diagnosed outcomes or procedures with associated ICD-10/OPCS codes and incident primary care outcomes with associated READ codes

| **Disease** | **ICD-10 codes** | **Read v2 codes** | **Read v3 (CTV3) codes** |
| --- | --- | --- | --- |
| Dementia | F00\|F01\|F02\|F03\|G30 | 1461.\|66h..\|6AB..\|8CMZ.\|9hD..\|9hD0.\|9hD1.\|9Ou..\|9Ou1.\|9Ou2.\|9Ou3.\|9Ou4.\|9Ou5.\|E00..\|E000.\|E001.\|E0010\|E0011\|E0012\|E0013\|E001z\|E002.\|E0020\|E0021\|E002z\|E003.\|E004.\|E0040\|E0041\|E0042\|E0043\|E004z\|E00y.\|E00z.\|E041.\|Eu00.\|Eu000\|Eu001\|Eu002\|Eu00z\|Eu01.\|Eu010\|Eu011\|Eu012\|Eu013\|Eu01y\|Eu01z\|Eu02z\|Eu041\|F110.\|F1100\|F1101\|Fyu30\|ZS7C5 | 1461.\|XaMJC\|XaMGF\|XaaBZ\|XaLFf\|XaLFo\|XaLFp\|XaMFy\|XaMG0\|XaMGG\|XaMGI\|XaMGJ\|XaMGK\|XE1Xr\|X00R2\|X002w\|E000.\|E001.\|E0010\|E0011\|E0012\|E0013\|E001z\|E002.\|E0020\|E0021\|E002z\|E003.\|XE1Xs\|Xa0lH\|E0040\|E0041\|E0042\|E0043\|E004z\|XE1Xt\|X00R0\|E00z.\|E041.\|Eu00.\|X002x\|X0030\|Eu002\|Eu00z\|X003R\|X003T\|X003V\|Eu01y\|Eu01z\|XE1Z6\|Eu02z\|Xa1GB\|Eu041\|F110.\|XaIKB\|XaIKC\|Fyu30 |
| Liver fibrosis or cirrhosis | K74* | J61y4\|J6160\|J616.\|J615.\|J615z\|Jyu71\|G8522 | X306R\|J61y3\|X307W\|X307Z\|J616.\|J6160\|J616z\|J61..\|J615.\|J6153\|J6155\|J615y\|J615z\|Jyu71\|X307L\|X307M\|X307O\|X307Q\|X307R\|XE0b5\|XE0bA\|XaBM6 |
| Liver cancer | C22 | B1503\|B15..\|B151.\|B151z\|Byu11\|B150.\|B1500\|B152. | B150.\|X78Oz\|Xa97q\|XE1xp\|B1500\|B1503\|XM1FE\|B1513\|B151z\|B150.\|B150z\|B152.\|X78Oz\|Xa97q\|XE1xp |
| Non-alcoholic fatty liver disease (NAFLD) | K76.0 | J61y.\|J61yz\|J61y1\|J61y9\|J61y9 | J61y.\|J61yz\|J61y1\| X307v |
| Osteoarthritis | M15.0\|M15.1\|M15.2\|M15.9\|M16.0\|M16.1\|M17.0\|M17.1\|M18.0\|M18.1\|M19.0 | 14G2.\|2G26.\|7P204\|N05..\|N050.\|N0500\|N0501\|N0502\|N0503\|N0504\|N0505\|N0506\|N0507\|N050z\|N051.\|N0510\|N0511\|N0512\|N0513\|N0514\|N0515\|N0516\|N0517\|N0518\|N0519\|N051A\|N051B\|N051C\|N051D\|N051E\|N051F\|N051G\|N051z\|N052.\|N0520\|N0521\|N0522\|N0523\|N0524\|N0525\|N0526\|N0527\|N0528\|N052z\|N053.\|N0530\|N0531\|N0532\|N0533\|N0534\|N0535\|N0536\|N0537\|N0538\|N0539\|N053z\|N054.\|N0540\|N0541\|N0542\|N0544\|N0545\|N0546\|N0547\|N0548\|N0549\|N054z\|N05z.\|N05z0\|N05z1\|N05z4\|N05z5\|N05z6\|N05z7\|N05z8\|N05z9\|N05zA\|N05zB\|N05zC\|N05zD\|N05zE\|N05zF\|N05zG\|N05zH\|N05zJ\|N05zK\|N05zL\|N05zM\|N05zN\|N05zP\|N05zQ\|N05zR\|N05zS\|N05zT\|N05zU\|N05zz\|Nyu2.\|Nyu20\|Nyu21\|Nyu22\|Nyu24\|Nyu25\|Nyu27\|Nyu28\|Nyu29\|Nyu2D\|Nyu2E | 14G2.\|2G26.\|XaLsk\|XE1DV\|N050.\|N0500\|XE1DW\|X76G5\|XM05w\|N0502\|N0503\|X7038\|N0505\|N0506\|N0507\|N050z\|N051.\|N0510\|N0511\|N0512\|N0513\|N0514\|N0515\|N0516\|N0517\|N0518\|N0519\|N051A\|N051B\|N051C\|XaEGd\|XaEGe\|XaEGf\|X703A\|N051z\|N052.\|N0520\|N0521\|N0522\|N0523\|N0524\|XE1DX\|X7043\|N0526\|N0527\|N0528\|N052z\|N053.\|N0530\|N0531\|N0532\|N0533\|N0534\|XE1DY\|N05zJ\|XE1DZ\|N0536\|X703L\|XaYQD\|N0537\|N0538\|N0539\|N053z\|N054.\|N0540\|N0541\|N0542\|N0544\|N0545\|N0546\|N0547\|N0548\|N0549\|N054z\|XE1Da\|N05z0\|N05z1\|XE1Dd\|X7035\|X7034\|XE1De\|XE1Df\|N05zL\|XE1Dg\|X7031\|X7030\|X702z\|N05z8\|N05z9\|N05zA\|N05zB\|N05zC\|N05zD\|N05zE\|N05zF\|N05zG\|N05zH\|N05zK\|N05zM\|N05zN\|N05zP\|N05zQ\|N05zR\|N05zS\|N05zT\|N05zU\|Nyu2.\|Nyu20\|Nyu21\|Nyu22\|Nyu24\|XE1GT\|Nyu27\|XE1GU\|Nyu29\|Nyu2D\|XE1GV |
| Type 2 diabetes (TD2) | E11 | C109.\|C1090\|C1091\|C1092\|C1094\|C1095\|C1096\|C1097\|C109A\|C109B\|C109C\|C109D\|C109E\|C109F\|C109G\|C109H\|C109J\|C109K\|C10F.\|C10F0\|C10F1\|C10F2\|C10F3\|C10F4\|C10F5\|C10F6\|C10F7\|C10F9\|C10FA\|C10FB\|C10FC\|C10FD\|C10FE\|C10FF\|C10FG\|C10FH\|C10FJ\|C10FK\|C10FL\|C10FM\|C10FN\|C10FP\|C10FQ\|C10FR | X40J5\|C1090\|C1091\|C1092\|C1094\|C1095\|C1096\|C1097\|XaEnp\|XaEnq\|XaF05\|XaFWI\|XaFmA\|XaFn7\|XaFn8\|XaFn9\|X40J6\|XaIrf\|C1093\|XaELQ\|XaIzQ\|XaIzR\|C1011\|C1031\|XaJQp\|XaKyX |
|  |  |  |  |
| **Procedure** | **OPCS Codes** |  |  |
| Ankle replacement | O32\|O320\|O321\|O322\|O323\|O324\|O325 | N/A | N/A |
| Hip replacement | W37\|W370\|W371\|W372\|W373\|W374\|W38\|W380\|W381\|W382\|W383\|W384\|W46\|W460\|W461\|W462\|W463\|W47\|W470\|W471\|W472\|W473\|W93\|W930\|W931\|W932\|W933\|W94\|W940\|W941\|W942\|W943\|O171\|O172\|O173\|W580\|W581\|W582 | N/A | N/A |
| Knee replacement | O18*\|W40*\|W41*\|W42* | N/A | N/A |
| Shoulder replacement | O06\|O060\|O061\|O062\|O063\|O068\|O069\|O07\|O070\|O071\|O072\|O073\|O078\|O079\|O08\|O080\|O081\|O082\|O083\|O084\|O088\|O089\|O09\|O091\|O098\|O099\|O10\|O101\|O108\|O109 | N/A | N/A |

Abbreviations: ICD-10 = International Classification of Diseases 10th revision codes; OPCS-4 = OPCS Classification of Interventions and Procedures version 4. Joint replacement surgery variable includes a diagnosis of hip, knee, ankle, or shoulder replacement.

Read codes as used in previous publications for liver fibrosis or cirrhosis/liver cancer^8^, NAFLD^9^ and dementia/osteoarthritis/T2D^10^.

*Excludes alcoholic fibrosis (K70.2) or cirrhosis (K70.3) of liver.

## **eTable 2.** Baseline characteristics of male and female p.C282Y homozygotes and those without *HFE* p.C282Y or p.H63D genotypes within UK Biobank

|  | Male p.C282Y homozygotes | Female p.C282Y homozygotes | Males without p.C282Y & p.H63D genotypes | Females without p.C282Y & p.H63D genotypes |
| --- | --- | --- | --- | --- |
| Total participants, n | 1,297 | 1,602 | 122,601 | 145,406 |
| Participants with primary care records, n (%) | 613 (47.3) | 773 (48.3) | 56,327 (45.9) | 67,770 (46.6) |
| Diagnosed hemochromatosis, n (%) | 157 (12.1) | 54 (3.4) | 29 (0.02) | 8 (0.01) |
| Mean age, years (SD) | 56.84 (8.2) | 56.92 (8.0) | 56.99 (8.1) | 56.62 (7.9) |
| WHR, (SD) | 0.94 (0.1) | 0.82 (0.1) | 0.94 (0.1) | 0.82 (0.1) |
| WHR ≥0.96, n (%) | 440 (33.92) | . | 41,824 (34.11) | . |
| WHR ≥0.85, n (%) | . | 480 (29.96) | . | 44,342 (30.50) |
| BMI(kg/m^2^), (SD) | 27.63 (4.1) | 26.92 (5.1) | 27.85 (4.2) | 27.02 (5.2) |
| Underweight (<18.5kg/m^2^), n (%) | 5 (0.39) | 13 (0.81) | 279 (0.23) | 1,071 (0.74) |
| Normal (18.5-24.9kg/m^2^), n (%) | 335 (25.87) | 647 (40.46) | 30,417 (24.85) | 57,299 (39.45) |
| Overweight (25-29.9kg/m^2^), n (%) | 662 (51.12) | 589 (36.84) | 60,388 (49.33) | 53,329 (36.71) |
| Obese (≥30kg/m^2^), n (%) | 293 (22.63) | 350 (21.89) | 31,320 (25.59) | 33,553 (23.10) |
| FIB-4 Score |  |  |  |  |
| Advanced fibrosis likely (>2.67), n (%) | 56 (4.67) | 37 (2.51) | 3,510 (3.08) | 1,850 (1.37) |
| NAFLD, n (%) | <5 | <5 | 163 (0.13) | 125 (0.09) |
| HbA1c (mmol/mol), (SD) | 39.92 (7.77) | 33.66 (5.42) | 36.52 (7.40) | 35.80 (5.65) |
| Viral hepatitis, n (%) | 7 (0.54) | 7 (0.44) | 751 (0.61) | 708 (0.49) |
| Alcoholic liver disease, n (%) | 6 (0.46) | 0 (0.00) | 203 (0.17) | 53 (0.04) |
| Alcohol intake frequency |  |  |  |  |
| Never, n (%) | 82 (6.32) | 132 (8.24) | 6,234 (5.08) | 11,730 (8.06) |
| Low, n (%) | 192 (14.80) | 440 (27.47) | 18,928 (15.42) | 39,483 (27.12) |
| Moderate, n (%) | 355 (27.37) | 441 (27.55) | 31,953 (26.03) | 38,442 (26.40) |
| High, n (%) | 668 (51.50) | 589 (42.53) | 65,624 (53.47) | 55,947 (38.42) |
| Ever smoked, n (%) | 694 (53.72) | 681 (42.51) | 63,190 (51.62) | 60,075 (41.38) |
| Highest educational qualification |  |  |  |  |
| None, n (%) | 264 (20.59) | 283 (17.85) | 21,472 (17.66) | 25,180 (17.44) |
| CSEs, n (%) | 48 (3.74) | 61 (3.85) | 4,109 (3.38) | 5,751 (3.98) |
| GCSEs/O-levels, n (%) | 138 (10.76) | 231 (14.57) | 12,439 (10.23) | 22,964 (15.91) |
| A-levels/NVQ/HND/HNC, n (%) | 262 (20.44) | 267 (16.85) | 25,344 (20.84) | 23,560 (16.32) |
| Professional qualification, n (%) | 173 (13.49) | 289 (18.23) | 17,505 (14.39) | 22,152 (15.35) |
| University degree, n (%) | 397 (30.97) | 454 (28.64) | 40,747 (33.50) | 44,751 (31.00) |
| Upper quartile Townsend index, n (%) | 350 (27.01) | 429 (26.76) | 31,082 (25.33) | 35,675 (24.51) |
| Upper quartile MET minutes per week, n (%) | 295 (26.84) | 276 (21.87) | 27,591 (26.52) | 26,778 (23.54) |

Male and female p.C282Y homozygous participants (n=2,899) and participants without *HFE* p.C282Y or p.H63D mutations (n=268,007) genetically similar to the 1000 genome project European Ancestry superpopulation (‘EUR-like’)^11^, the categorization and detailed definition of this population has previously been defined^12^, with *HFE* genotypic data available in the UK Biobank. Numbers presented are mean (SD) for continuous variables and n (%) for categorical variables. Cut-offs for WHR were ≥0.96 for men and ≥0.85 for women. Abbreviations: SD, standard deviation; WHR, waist-hip-ratio; NAFLD, non-alcoholic fatty liver disease; FIB-4, Fibrosis-4; BMI, Body Mass Index; HbA1c, hemoglobin A1c; A-level, Advanced level; CSEs, Certificate of Secondary Education; GCSEs, General Certificate of Secondary Education; HNC, Higher National Certificate; HND, Higher National Diploma; MET, Metabolic Equivalent Task; NVC, National Vocational Certification; NVQ, National Vocational Qualification; O-level, Ordinary level.

## **eTable 3**. Risk of incident hospital diagnoses in male p.C282Y homozygous UK Biobank participants by WHR

|  |  |  |  |  | Model 1 | | Model 2 | |
| --- | --- | --- | --- | --- | --- | --- | --- | --- |
| Incident outcomes | **WHR** | **Incident cases,**  **n (%)** | **Diagnosed with hemochromatosis, by end of follow-up (%)** |  | **Hazard Ratio (95% CI)** | **p-value** | **Hazard Ratio (95% CI)** | **p-value** |
| Liver fibrosis or cirrhosis | Normal | 12 (1.42) | 12 (100.00) |  | 1 | 8.4*10^-5^ | 1 | 3.1*10^-3^ |
|  | High | 24 (5.61) | 23 (95.83) |  | 4.13 (2.04-8.39) |  | 3.37 (1.51-7.53) |  |
| Liver cancer | Normal | 12 (1.40) | 10 (83.33) |  | 1 | 0.01 | 1 | 0.046 |
|  | High | 19 (4.34) | 16 (84.21) |  | 2.57 (1.24-5.33) |  | 2.61 (1.02-6.67) |  |
| NAFLD | Normal | 22 (2.57) | 21 (95.45) |  | 1 | 1.8*10^-6^ | 1 | 1.9*10^-4^ |
|  | High | 35 (7.97) | 26 (74.29) |  | 3.86 (2.22-6.72) |  | 3.25 (1.75-6.03) |  |
| Type 2 diabetes | Normal | 38 (4.54) | 16 (42.11) |  | 1 | 5.3*10^-12^ | 1 | 5.9*10^-8^ |
|  | High | 77 (19.01) | 34 (44.16) |  | 4.03 (2.71-5.98) |  | 3.55 (2.25-5.62) |  |
| Dementia | Normal | 21 (2.45) | 5 (23.81) |  | 1 | 0.31 | 1 | 0.52 |
|  | High | 19 (4.34) | 10 (52.63) |  | 1.39 (0.74-2.61) |  | 1.29 (0.59-2.81) |  |
| Osteoarthritis | Normal | 34 (4.52) | 18 (52.94) |  | 1 | 0.17 | 1 | 0.68 |
|  | High | 27 (7.52) | 11 (40.74) |  | 1.44 (0.86-2.40) |  | 1.14 (0.62-2.10) |  |
| Joint replacement surgery | Normal | 89 (11.03) | 39 (43.82) |  | 1 | 0.85 | 1 | 0.56 |
|  | High | 55 (13.38) | 19 (34.55) |  | 1.03 (0.74-1.45) |  | 0.89 (0.59-1.33) |  |

Risk of incident outcomes (excluding prevalent disease at baseline) presented in male p.C282Y homozygotes (n=1,297) with a high WHR (≥0.96) compared to those with a normal WHR. Model 1 adjusted for age & principal components 1-10. Model 2 additionally adjusted for alcohol intake, smoking status, physical activity, education, Townsend deprivation index, baseline diagnosis of viral hepatitis and baseline diagnosis of alcoholic liver disease (n=1,087 due to missing covariate data). Joint replacement surgery variable includes a diagnosis of hip, knee, ankle, or shoulder replacement. Abbreviations: WHR, waist-to-hip ratio; NAFLD, non-alcoholic fatty liver disease.

## **eTable 4.** Cumulative incidence of outcomes from ages 40-80 years by WHR status in p.C282Y homozygous male and females and those without p.C282Y or p.H63D genotypes variants

|  | **p.C282Y homozygotes** | | | | | | | | | | | | | | **No p.C282Y or p.H63D genotypes** | | | | | | | | | | | | | |
| --- | --- | --- | --- | --- | --- | --- | --- | --- | --- | --- | --- | --- | --- | --- | --- | --- | --- | --- | --- | --- | --- | --- | --- | --- | --- | --- | --- | --- |
|  | **Normal WHR** | | | |  | | | | | **High WHR** | | | | | **Normal WHR** | | | |  | | | | | **High WHR** | | | | |
|  | **%** | | **(95% CI)** | | |  | | | ***%*** | | | **(95% CI)** | | **%** | | | **(95% CI)** | | |  | | | ***%*** | | | **(95% CI)** | |  |
| **MALES** |  |  | |  | | |  |  | | |  | |  |  | |  | |  | | |  |  | | |  | |  |  |
| Liver fibrosis or cirrhosis | 3.9 | 1.9 | | 7.6 | | |  | 15.0 | | | 9.8 | | 22.6 | 0.8 | | 0.7 | | 1.0 | | |  | 2.0 | | | 1.8 | | 2.3 |  |
| Liver cancer | 3.6 | 1.9 | | 6.6 | | |  | 9.2 | | | 5.7 | | 14.6 | 0.6 | | 0.5 | | 0.7 | | |  | 1.1 | | | 0.9 | | 1.2 |  |
| NAFLD | 6.4 | 4.2 | | 9.7 | | |  | 20.9 | | | 14.8 | | 29.1 | 2.3 | | 2.0 | | 2.5 | | |  | 6.5 | | | 6.0 | | 7.1 |  |
| Type 2 diabetes | 11.8 | 8.4 | | 16.4 | | |  | 45.0 | | | 35.8 | | 55.3 | 11.0 | | 10.6 | | 11.4 | | |  | 34.1 | | | 33.2 | | 34.9 |  |
|  |  |  | |  | | |  |  | | |  | |  |  | |  | |  | | |  |  | | |  | |  |  |
| **FEMALES** |  |  | |  | | |  |  | | |  | |  |  | |  | |  | | |  |  | | |  | |  |  |
| Liver fibrosis or cirrhosis | 0.6 | 0.2 | | 1.8 | | |  | 4.6 | | | 2.5 | | 8.2 | 0.5 | | 0.5 | | 0.6 | | |  | 1.6 | | | 1.4 | | 1.8 |  |
| NAFLD | 2.7 | 1.4 | | 5.3 | | |  | 8.9 | | | 5.7 | | 13.7 | 1.8 | | 1.7 | | 2.0 | | |  | 6.6 | | | 6.2 | | 7.1 |  |

WHR cut-offs were ≥0.96 for men and ≥0.85 for women. Abbreviations: CI, confidence intervals; WHR, waist-to-hip ratio; NAFLD, non-alcoholic fatty liver disease.

## **eTable 5.** Risk of incident hospital diagnoses in male p.C282Y homozygous UK Biobank participants by BMI status

|  |  |  | **Model 1** | | **Model 2** | |
| --- | --- | --- | --- | --- | --- | --- |
| **Incident outcomes** | **BMI categories** | **Incident cases,** | **Hazard Ratio (95% CI)** | **p-value** | **Hazard Ratio (95% CI)** | **p-value** |
|  |  | **n (%)** |  |  |  |  |
| **Liver fibrosis or cirrhosis** | Underweight | 0 (0.00) | N/A | N/A | N/A | N/A |
|  | Normal | 7 (2.12) | 1 | 1 | 1 | 1 |
|  | Overweight | 16 (2.44) | 1.11 (0.45-2.73) | 0.81 | 0.95 (0.37-2.39) | 0.91 |
|  | Obese | 13 (4.56) | 2.18 (0.87-5.50) | 0.10 | 1.28 (0.45-3.61) | 0.65 |
| **Liver cancer** | Underweight | 0 (0.00) | N/A | N/A | N/A | N/A |
|  | Normal | 7 (2.10) | 1 | 1 | 1 | 1 |
|  | Overweight | 16 (2.42) | 1.01 (0.41-2.49) | 0.99 | 2.08 (0.61-7.14) | 0.24 |
|  | Obese | 8 (2.74) | 1.28 (0.46-3.56) | 0.64 | 1.91 (0.45-8.11) | 0.38 |
| **NAFLD** | Underweight | 0 (0.00) | N/A | N/A | N/A | N/A |
|  | Normal | 8 (2.39) | 1 | 1 | 1 | 1 |
|  | Overweight | 21 (3.18) | 1.42 (0.63-3.24) | 0.40 | 1.73 (0.68-4.40) | 0.25 |
|  | Obese | 28 (9.59) | 4.52 (2.05-9.97) | 0.0002 | 4.55 (1.80-11.48) | 1.4*10^-3^ |
| **Type 2 diabetes** | Underweight | 0 (0.00) | N/A | N/A | N/A | N/A |
|  | Normal | 8 (2.45) | 1 | 1 | 1 | 1 |
|  | Overweight | 50 (7.82) | 3.03 (1.43-6.42) | 0.004 | 4.90 (1.75-13.74) | 2.5*10^-3^ |
|  | Obese | 57 (21.19) | 9.37 (4.46-19.69) | 3.6*10^-9^ | 15.73 (5.60-44.19) | 1.7*10^-7^ |
| **Dementia** | Underweight | 0 (0.00) | N/A | N/A | N/A | N/A |
|  | Normal | 12 (3.58) | 1 | 1 | 1 | 1 |
|  | Overweight | 21 (3.17) | 0.66 (0.32-1.37) | 0.27 | 0.78 (0.32-1.92) | 0.59 |
|  | Obese | 7 (2.41) | 0.60 (0.23-1.56) | 0.29 | 0.71 (0.23-2.25) | 0.56 |
| **Osteoarthritis** | Underweight | 0 (0.00) | N/A | N/A | N/A | N/A |
|  | Normal | 15 (5.03) | 1 | 1 | 1 | 1 |
|  | Overweight | 32 (5.67) | 0.98 (0.53-1.82) | 0.95 | 0.77 (0.39-1.50) | 0.44 |
|  | Obese | 14 (5.69) | 1.06 (0.51-2.21) | 0.87 | 0.71 (0.31-1.64) | 0.42 |
| **Joint replacement surgery** | Underweight | 0 (0.00) | N/A | N/A | N/A | N/A |
|  | Normal | 25 (7.79) | 1 | 1 | 1 | 1 |
|  | Overweight | 79 (12.72) | 1.46 (0.93-2.29) | 0.10 | 1.27 (0.78-2.06) | 0.34 |
|  | Obese | 39 (14.44) | 1.81 (1.09-2.99) | 0.02 | 1.42 (0.81-2.50) | 0.22 |

Risk of incident outcomes (excluding prevalent disease at baseline) presented in male p.C282Y homozygotes by BMI (n=1,295). BMI was categorized as underweight (<18.5kg/m^2^), normal (reference group: 18.5-24.9kg/m^2^), overweight (25-29.9kg/m^2^) and obese (≥30kg/m^2^). Model 1 adjusted for age & principal components 1-10. Model 2 adjusted for alcohol intake, smoking status, physical activity, education, Townsend deprivation index, baseline diagnosis of viral hepatitis and baseline diagnosis of alcoholic liver disease (n=1,086 due to missing covariate data). Joint replacement surgery variable includes a diagnosis of hip, knee, ankle, or shoulder replacement. Abbreviations: BMI, body mass index; NAFLD, non-alcoholic fatty liver disease.

## **eTable 6:** Risk of incident hospital diagnoses in female p.C282Y homozygous UK Biobank participants by WHR

|  |  |  |  |  | |  | **Model 1** | | | **Model 2** | | |
| --- | --- | --- | --- | --- | --- | --- | --- | --- | --- | --- | --- | --- |
| **Incident outcomes** | **WHR** | **Incident cases,**  **n (%)** | **Diagnosed with hemochromatosis, by end of follow-up (%)** | | **Hazard Ratio (95% CI)** | | | **p-value** | **Hazard Ratio (95% CI)** | | **p-value** |  |
|  |  |  |  |  |  |  |  |  |  |  |  |  |
| **Liver fibrosis or cirrhosis** | Normal | <5 | <5 | | 1 | | | 8.0*10^-4^ | 1 | | 2.5*10^-3^ |  |
|  | High | 11 (2.30) | 9 (81.82) | | 9.17 (2.51-33.50) | | |  | 9.51 (2.21-40.87) | |  |  |
| **NAFLD** | Normal | 12 (1.07) | 7 (58.33) | | 1 | | | 1.2*10^-5^ | 1 | | 9.3*10^-5^ |  |
|  | High | 21 (4.38) | 11 (52.38) | | 5.17 (2.48-10.78) | | |  | 5.27 (2.29-12.14) | |  |  |
| **Osteoarthritis** | Normal | 48 (4.92) | 12 (25.00) | | 1 | | | 1 | 1 | | 0.51 |  |
|  | High | 22 (5.61) | 9 (40.91) | | 1.00 (0.60-1.67) | | |  | 0.81 (0.43-1.53) | |  |  |
| **Joint replacement surgery** | Normal | 95 (8.81) | 32 (33.68) | | 1 | | | 0.03 | 1 | | 0.49 |  |
|  | High | 63 (13.97) | 23 (36.51) | | 1.42 (1.03-1.96) | | |  | 1.15 (0.77-1.72) | |  |  |

Risk of incident outcomes (excluding prevalent disease at baseline) presented in female p.C282Y homozygotes (n=1,602) with a high WHR (≥0.85) compared to those with a normal WHR. Model 1 adjusted for age & principal components 1-10, hazard ratios (95%CI). Model 2 additionally adjusted for alcohol intake, smoking status, physical activity, education, Townsend deprivation index, baseline diagnosis of viral hepatitis and baseline diagnosis of alcoholic liver disease (n=1,248 due to missing covariate data). Joint replacement surgery variable includes a diagnosis of hip, knee, ankle, or shoulder replacement. Abbreviations: WHR, waist-to-hip ratio; NAFLD, non-alcoholic fatty liver disease.

## **eTable 7.** Risk of incident hospital diagnoses in female p.C282Y homozygous UK Biobank participants by BMI status

|  |  |  | **Model 1** | | **Model 2** | |  |
| --- | --- | --- | --- | --- | --- | --- | --- |
| **Incident outcomes** | **BMI categories** | **Incident cases**, **n (%)** | **Hazard Ratio (95% CI)** | **p-value** | **Hazard Ratio (95% CI)** | **p-value** |  |
|  |  |  |  |  |  |  |  |
|  | Underweight | 0 (0.00) | N/A | N/A | N/A | N/A |  |
| **Liver fibrosis or cirrhosis** | Normal | <5 | 1 | 1 | 1 | 1 |  |
|  | Overweight | 6 (1.02) | 2.26 (0.56-9.15) | 0.25 | 1.92 (0.43-8.65) | 0.39 |  |
|  | Obese | 5 (1.43) | 3.35 (0.78-14.35) | 0.10 | 2.52 (0.41-15.58) | 0.32 |  |
|  | Underweight | 0 (0.00) | N/A | N/A | N/A | N/A |  |
| **NAFLD** | Normal | 5 (0.77) | 1 | 1 | 1 | 1 |  |
|  | Overweight | 15 (2.56) | 3.34 (1.21-9.21) | 0.02 | 3.21 (1.05-9.87) | 0.04 |  |
|  | Obese | 13 (3.71) | 5.20 (1.84-14.69) | 0.002 | 3.90 (1.16-13.03) | 0.03 |  |
|  | Underweight | 0 (0.00) | N/A | N/A | N/A | N/A |  |
| **Osteoarthritis** | Normal | 22 (3.81) | 1 | 1 | 1 | 1 |  |
|  | Overweight | 31 (6.19) | 1.45 (0.83-2.51) | 0.19 | 1.53 (0.82-2.88) | 0.18 |  |
|  | Obese | 17 (6.16) | 1.35 (0.71-2.55) | 0.36 | 0.91 (0.38-2.17) | 0.82 |  |
|  | Underweight | 0 (0.00) | N/A | N/A | N/A | N/A |  |
| **Joint replacement surgery** | Normal | 44 (6.97) | 1 | 1 | 1 | 1 |  |
|  | Overweight | 67 (12.03) | 1.59 (1.08-2.33) | 0.02 | 1.74 (1.11-2.71) | 0.02 |  |
|  | Obese | 47 (14.37) | 1.92 (1.27-2.90) | 0.002 | 1.85 (1.09-3.15) | 0.02 |  |

Risk of incident outcomes (excluding prevalent disease at baseline) presented in female p.C282Y homozygotes by BMI (n=1,599). BMI was categorized as underweight (<18.5kg/m^2^), normal (reference group: 18.5-24.9kg/m^2^), overweight (25-29.9kg/m^2^) and obese (≥30kg/m^2^). Model 1 adjusted for age & principal components 1-10. Model 2 adjusted for alcohol intake, smoking status, physical activity, education, Townsend deprivation index, baseline diagnosis of viral hepatitis and baseline diagnosis of alcoholic liver disease (n=1,247 due to missing covariate data). Joint replacement surgery variable includes a diagnosis of hip, knee, ankle, or shoulder replacement. Abbreviations: BMI, body mass index; NAFLD, non-alcoholic fatty liver disease.

## **eTable 8.** Interaction analysis for multiplicative effect of *HFE* p.C282Y homozygous males and high WHR on risk of incident hospital diagnoses compared to males without *HFE* p.C282Y or p.H63D genotypes and normal WHR

|  | Hazard ratios | 95% CI | | p-value |
| --- | --- | --- | --- | --- |
| Liver fibrosis or cirrhosis |  |  |  |  |
| p.C282Y homozygotes # WHR ≥0.96 | 1.39 | 0.68 | 2.84 | 0.36 |
| Liver cancer |  |  |  |  |
| p.C282Y homozygotes # WHR ≥0.96 | 1.32 | 0.62 | 2.81 | 0.46 |
| NAFLD |  |  |  |  |
| p.C282Y homozygotes # WHR ≥0.96 | 1.05 | 0.61 | 1.81 | 0.86 |
| Type 2 diabetes |  |  |  |  |
| p.C282Y homozygotes # WHR ≥0.96 | 1.19 | 0.81 | 0.76 | 0.38 |
| Osteoarthritis |  |  |  |  |
| p.C282Y homozygotes # WHR ≥0.96 | 1.01 | 0.61 | 1.69 | 0.96 |
| Joint replacement surgery |  |  |  |  |
| p.C282Y homozygotes # WHR ≥0.96 | 0.77 | 0.55 | 1.08 | 0.14 |
| Dementia |  |  |  |  |
| p.C282Y homozygotes # WHR ≥0.96 | 1.15 | 0.61 | 2.15 | 0.66 |

Risk of incident outcomes excluding prevalent disease at baseline. Multiplicative effect determined by ‘p-value’ <0.05. Joint replacement surgery variable includes a diagnosis of hip, knee, ankle, or shoulder replacement. Abbreviations: CI, confidence intervals; WHR, waist-to-hip ratio; NAFLD, non-alcoholic fatty liver disease.

## **eTable 9.** Interaction analysis for multiplicative effect of *HFE* p.C282Y homozygous females and high WHR on risk of incident hospital diagnoses compared to females without *HFE* p.C282Y or p.H63D genotypes and normal WHR

|  | **Hazard ratios** | **95% CI** | | **p-value** |
| --- | --- | --- | --- | --- |
| **Liver fibrosis or cirrhosis** |  |  |  |  |
| p.C282Y homozygotes # WHR ≥0.85 | 2.64 | 0.73 | 9.58 | 0.14 |
| **NAFLD** |  |  |  |  |
| p.C282Y homozygotes # WHR ≥0.85 | 1.11 | 0.54 | 2.27 | 0.78 |
| **Osteoarthritis** |  |  |  |  |
| p.C282Y homozygotes # WHR ≥0.85 | 0.69 | 0.41 | 1.14 | 0.15 |
| **Joint replacement surgery** |  |  |  |  |
| p.C282Y homozygotes # WHR ≥0.85 | 1.04 | 0.76 | 1.44 | 0.79 |

Risk of incident outcomes excluding prevalent disease at baseline. Multiplicative effect determined by ‘p-value’ <0.05. Joint replacement surgery variable includes a diagnosis of hip, knee, ankle, or shoulder replacement. Abbreviations: CI, confidence intervals; WHR, waist-to-hip ratio; NAFLD, non-alcoholic fatty liver disease.

## **eTable 10.** Interaction analysis for multiplicative effect of *HFE* p.C282Y homozygous males and BMI groups on risk of incident hospital diagnoses compared to males without *HFE* p.C282Y or p.H63D genotypes and normal BMI

|  | **Hazard ratio** | **95% CI** | | **p-value** |
| --- | --- | --- | --- | --- |
| **Liver fibrosis or cirrhosis** |  |  |  |  |
| p.C282Y homozygotes # underweight | N/A | N/A | N/A | N/A |
| p.C282Y homozygotes # overweight | 0.88 | 0.35 | 2.22 | 0.79 |
| p.C282Y homozygotes # obese | 0.57 | 0.22 | 1.48 | 0.25 |
| **Liver cancer** |  |  |  |  |
| p.C282Y homozygotes # underweight | N/A | N/A | N/A | N/A |
| p.C282Y homozygotes # overweight | 0.79 | 0.31 | 2.03 | 0.63 |
| p.C282Y homozygotes # obese | 0.49 | 0.17 | 1.41 | 0.18 |
| **NAFLD** |  |  |  |  |
| p.C282Y homozygotes # underweight | N/A | N/A | N/A | N/A |
| p.C282Y homozygotes # overweight | 0.58 | 0.25 | 1.34 | 0.20 |
| p.C282Y homozygotes # obese | 0.72 | 0.32 | 1.61 | 0.43 |
| **Type 2 diabetes** |  |  |  |  |
| p.C282Y homozygotes # underweight | N/A | N/A | N/A | N/A |
| p.C282Y homozygotes # overweight | 1.23 | 0.58 | 2.6 | 0.59 |
| p.C282Y homozygotes # obese | 1.22 | 0.58 | 2.58 | 0.59 |
| **Osteoarthritis** |  |  |  |  |
| p.C282Y homozygotes # underweight | N/A | N/A | N/A | N/A |
| p.C282Y homozygotes # overweight | 0.64 | 0.34 | 1.19 | 0.16 |
| p.C282Y homozygotes # obese | 0.39 | 0.19 | 0.82 | 0.01 |
| **Joint replacement surgery** |  |  |  |  |
| p.C282Y homozygotes # underweight | N/A | N/A | N/A | N/A |
| p.C282Y homozygotes # overweight | 0.9 | 0.57 | 1.42 | 0.65 |
| p.C282Y homozygotes # obese | 0.69 | 0.41 | 1.14 | 0.15 |
| **Dementia** |  |  |  |  |
| p.C282Y homozygotes # underweight | N/A | N/A | N/A | N/A |
| p.C282Y homozygotes # overweight | 0.77 | 0.37 | 1.57 | 0.47 |
| p.C282Y homozygotes # obese | 0.53 | 0.21 | 1.35 | 0.18 |

Risk of incident outcomes excluding prevalent disease at baseline. Multiplicative effect determined by ‘p-value’ <0.05. Joint replacement surgery variable includes a diagnosis of hip, knee, ankle, or shoulder replacement. BMI was categorized as underweight (<18.5kg/m^2^), normal (reference group: 18.5-24.9kg/m^2^), overweight (25-29.9kg/m^2^) and obese (≥30kg/m^2^). Abbreviations: CI, confidence intervals; WHR, waist-to-hip ratio; NAFLD, non-alcoholic fatty liver disease.

## **eTable 11.** Interaction analysis for multiplicative effect of *HFE* p.C282Y homozygous females and BMI groups on risk of incident hospital diagnoses compared to females without *HFE* p.C282Y or p.H63D genotypes and normal BMI

|  | **Hazard ratio** | **95% CI** | | **p-value** |
| --- | --- | --- | --- | --- |
| **Liver fibrosis or cirrhosis** |  |  |  |  |
| p.C282Y homozygotes # underweight | N/A | N/A | N/A | N/A |
| p.C282Y homozygotes # overweight | 1.28 | 0.31 | 5.22 | 0.73 |
| p.C282Y homozygotes # obese | 0.85 | 0.2 | 3.61 | 0.82 |
| **NAFLD** |  |  |  |  |
| p.C282Y homozygotes # underweight | N/A | N/A | N/A | N/A |
| p.C282Y homozygotes # overweight | 1.08 | 0.39 | 3.02 | 0.88 |
| p.C282Y homozygotes # obese | 0.61 | 0.22 | 1.74 | 0.36 |
| **Osteoarthritis** |  |  |  |  |
| p.C282Y homozygotes # underweight | N/A | N/A | N/A | N/A |
| p.C282Y homozygotes # overweight | 0.98 | 0.57 | 1.7 | 0.95 |
| p.C282Y homozygotes # obese | 0.59 | 0.31 | 1.12 | 0.10 |
| **Joint replacement surgery** |  |  |  |  |
| p.C282Y homozygotes # underweight | N/A | N/A | N/A | N/A |
| p.C282Y homozygotes # overweight | 1.03 | 0.7 | 1.51 | 0.89 |
| p.C282Y homozygotes # obese | 0.78 | 0.51 | 1.18 | 0.23 |

Risk of incident outcomes excluding prevalent disease at baseline. Multiplicative effect determined by ‘p-value’ <0.05. Joint replacement surgery variable includes a diagnosis of hip, knee, ankle, or shoulder replacement. BMI was categorized as underweight (<18.5kg/m^2^), normal (reference group: 18.5-24.9kg/m^2^), overweight (25-29.9kg/m^2^) and obese (≥30kg/m^2^). Abbreviations: CI, confidence intervals; WHR, waist-to-hip ratio; NAFLD, non-alcoholic fatty liver disease.

## **eTable 12.** Risk of incident hospital diagnoses in male p.C282Y homozygous UK Biobank participants by WHR, excluding those with a diagnosis of hemochromatosis at baseline

| Incident outcomes | WHR | Incident cases, n (%) | Hazard Ratio (95% CI) | p-value |
| --- | --- | --- | --- | --- |
| Liver fibrosis or cirrhosis | Normal | 9 (1.17) | 1 | 4.4*10^-4^ |
|  | High | 18 (4.86) | 4.38 (1.92-10.00) |  |
| Liver cancer | Normal | 7 (0.91) | 1 | 0.08 |
|  | High | 10 (2.70) | 2.46 (0.91-6.61) |  |
| NAFLD | Normal | 18 (2.34) | 1 | 1.1*10^-4^ |
|  | High | 24 (6.49) | 3.58 (1.87-6.84) |  |
| Type 2 diabetes | Normal | 34 (4.49) | 1 | 2.2*10^-9^ |
|  | High | 63 (18.21) | 3.68 (2.40-5.65) |  |
| Dementia | Normal | 18 (2.34) | 1 | 0.35 |
|  | High | 15 (4.08) | 1.40 (0.70-2.80) |  |
| Osteoarthritis | Normal | 29 (4.21) | 1 | 0.16 |
|  | High | 22 (7.14) | 1.49 (0.85-2.63) |  |
| Joint replacement surgery | Normal | 77 (10.43) | 1 | 0.50 |
|  | High | 49 (14.08) | 1.13 (0.79-1.63) |  |

Risk of incident outcomes (excluding prevalent disease and hemochromatosis at baseline) presented in male p.C282Y homozygotes (n=1,140) with a high WHR (≥0.96) compared to those with a normal WHR. Adjusted for age & principal components 1-10. Abbreviations: CI, confidence interval; WHR, waist-to-hip ratio; NAFLD, non-alcoholic fatty liver disease; Joint replacement surgery variable includes a diagnosis of hip, knee, ankle, or shoulder replacement.

## **eTable 13.** Risk of incident hospital diagnoses in female p.C282Y homozygous UK Biobank participants by WHR, excluding those with a diagnosis of hemochromatosis at baseline

| **Incident outcomes** | **WHR** | **Incident cases, n (%)** | **Hazard Ratio (95% CI)** | **p-value** |
| --- | --- | --- | --- | --- |
| **Liver fibrosis or cirrhosis** | Normal | <5 | 1 | 7.3*10^-4^ |
|  | High | 11 (2.38) | 9.30 (2.55-33.97) |  |
| **NAFLD** | Normal | 11 (1.01) | 1 | 3.0*10^-5^ |
|  | High | 19 (4.11) | 5.16 (2.39-11.14) |  |
| **Osteoarthritis** | Normal | 46 (4.84) | 1 | 0.89 |
|  | High | 22 (5.82) | 1.04 (0.62-1.74) |  |
| **Joint replacement surgery** | Normal | 88 (8.42) | 1 | 0.01 |
|  | High | 62 (14.25) | 1.52 (1.09-2.12) |  |

Risk of incident outcomes (excluding prevalent disease and hemochromatosis at baseline) presented in female p.C282Y homozygotes (n=1,548) with a high WHR (≥0.85) compared to those with a normal WHR. Adjusted for age & principal components 1-10. Abbreviations: CI, confidence interval; WHR, waist-to-hip ratio; NAFLD, non-alcoholic fatty liver disease; Joint replacement surgery variable includes a diagnosis of hip, knee, ankle, or shoulder replacement.

## **eTable 14**. Risk of outcomes using incident primary care and hospital diagnoses in male p.C282Y homozygous UK Biobank participants by WHR

| **Incident outcomes** | **WHR** | **Incident cases,** | **Hazard Ratio (95% CI)** | **p-value** |
| --- | --- | --- | --- | --- |
|  |  | **n (%)** |  |  |
| **Liver fibrosis or cirrhosis** | Normal | 6 (1.47) | 1 | 8.3*10^-4^ |
|  | High | 13 (6.88) | 5.73 (2.06-15.92) |  |
| **Liver cancer** | Normal | 9 (2.17) | 1 | 0.21 |
|  | High | 9 (4.59) | 1.89 (0.69-5.16) |  |
| **NAFLD** | Normal | 18 (4.36) | 1 | 6.6*10^-3^ |
|  | High | 17 (8.67) | 2.66 (1.31-5.41) |  |
| **Type 2 diabetes** | Normal | 18 (4.51) | 1 | 2.8*10^-4^ |
|  | High | 25 (15.15) | 3.20 (1.71-6.00) |  |
| **Dementia** | Normal | 11 (2.65) | 1 | 0.70 |
|  | High | 11 (5.58) | 1.19 (0.48-2.96) |  |
| **Osteoarthritis** | Normal | 37 (11.25) | 1 | 0.50 |
|  | High | 22 (15.60) | 1.21 (0.70-2.08) |  |

Additional primary care data available in a subset of male p.C282Y homozygotes (n=613) with a high WHR (≥0.96) compared to those with a normal WHR; adjusted for age & principal components 1-10. Abbreviations: WHR, waist-to-hip ratio. Joint replacement surgeries were not analyzed in primary care data as they were likely to be captured within the hospital OPCS-4 codes.

## **eTable 15**. Risk of outcomes using incident primary care and hospital diagnoses in female p.C282Y homozygous UK Biobank participants by WHR

| **Incident outcomes** | **WHR** | **Incident cases,** | **Hazard Ratio (95% CI)** | **p-value** |
| --- | --- | --- | --- | --- |
|  |  | **n (%)** |  |  |
| **Liver fibrosis or cirrhosis** | Normal | <5 | 1 | 6.2*10^-3^ |
|  | High | 6 (2.86) | 10.70 (1.96-58.47) |  |
| **NAFLD** | Normal | 8 (1.43) | 1 | 4.5*10^-4^ |
|  | High | 13 (6.16) | 5.28 (2.08-13.38) |  |
| **Osteoarthritis** | Normal | 56 (12.36) | 1 | 0.59 |
|  | High | 20 (13.07) | 1.16 (0.68-1.95) |  |

Additional primary care data available in a subset of female p.C282Y homozygotes (n=773) with a high WHR (≥0.85) compared to those with a normal WHR; adjusted for age & principal components 1-10. Abbreviations: WHR, waist-to-hip ratio. Joint replacement surgeries were not analyzed in primary care data as they were likely to be captured within the hospital OPCS-4 codes.

# **FIGURES**

## **eFigure 1.** Spline regression for the association between WHR and risk of incident outcomes in all male UK Biobank participants

a) Liver fibrosis or cirrhosis


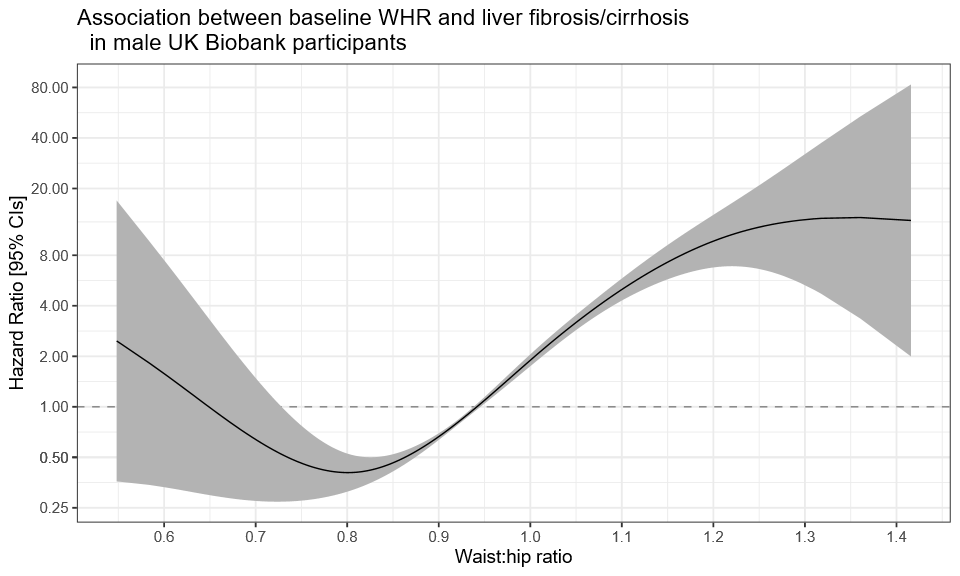


b) Liver cancer


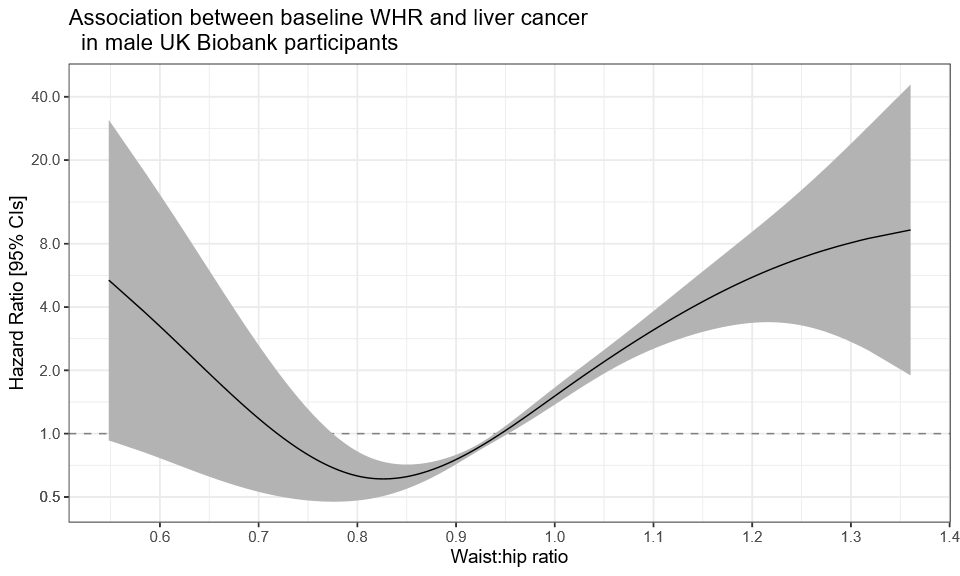


c) NAFLD


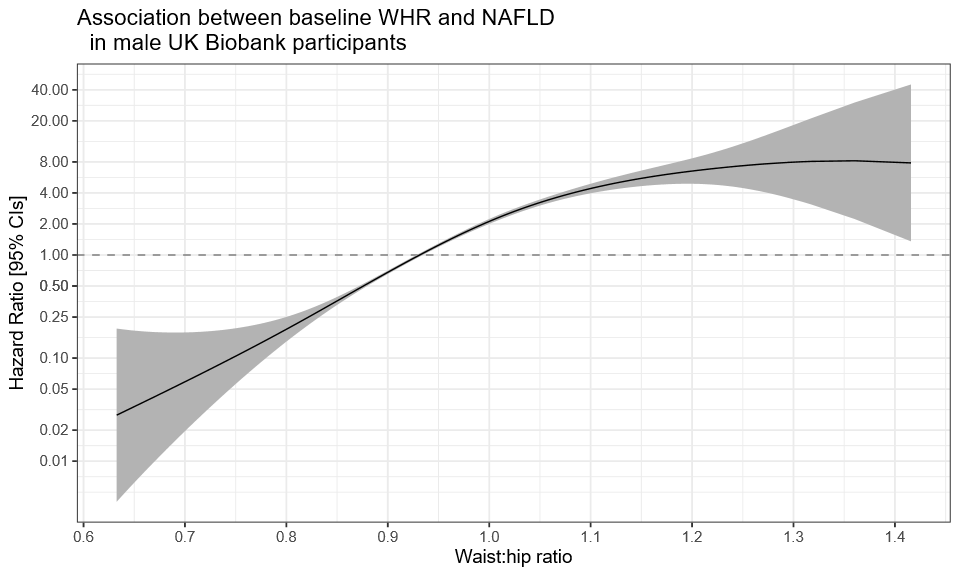


d) Type 2 diabetes


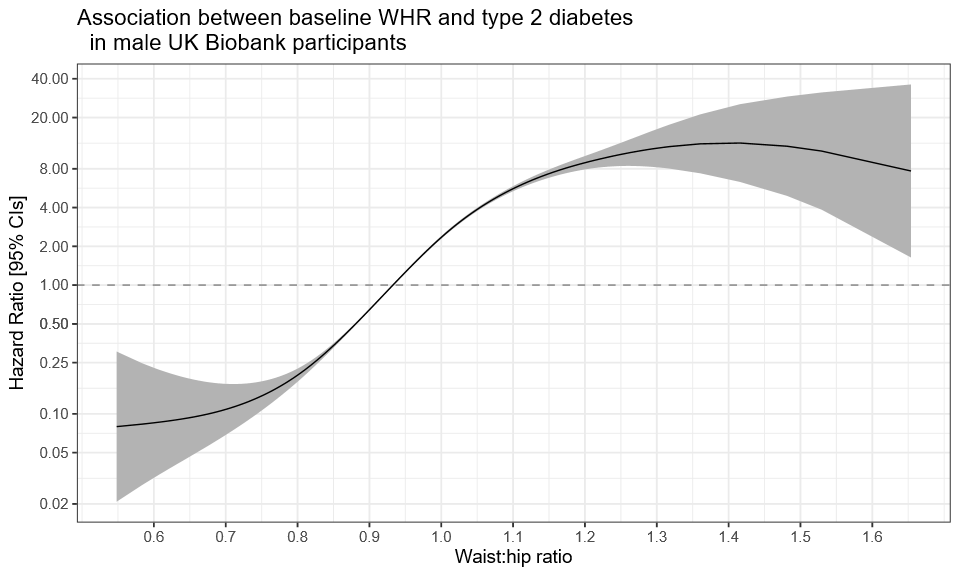


Spline point regression plots in male UK Biobank participants (n=205,992). Graphs a) = liver fibrosis/cirrhosis, b) = liver cancer, c) = NAFLD and d) = type 2 diabetes. Each model was adjusted for age. Abbreviations: CI, confidence interval; WHR, waist-to-hip ratio; NAFLD, non-alcoholic fatty liver disease.

## **eFigure 2.** Spline regression for the association between WHR and risk of incident outcomes in all female UK Biobank participants

1. Liver fibrosis/cirrhosis


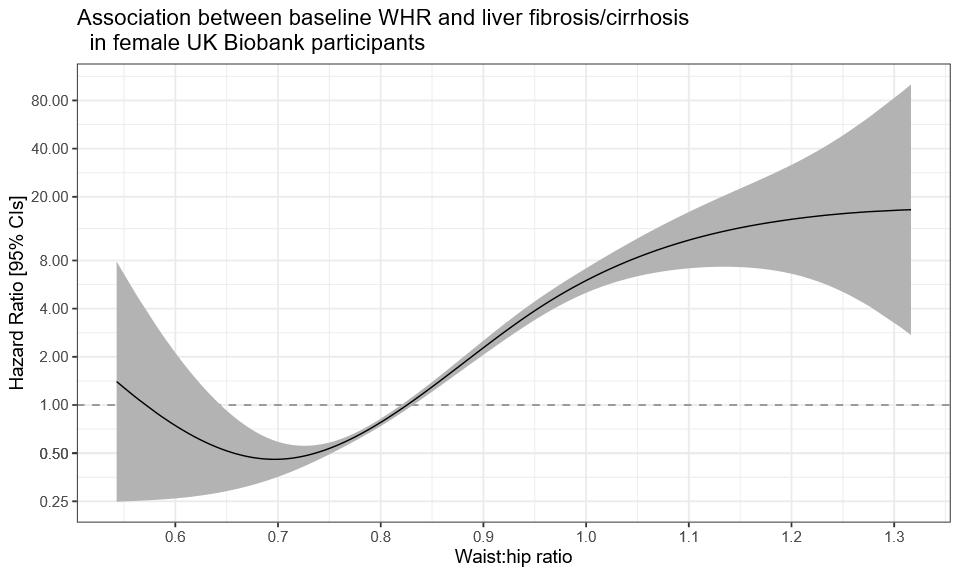


b) NAFLD
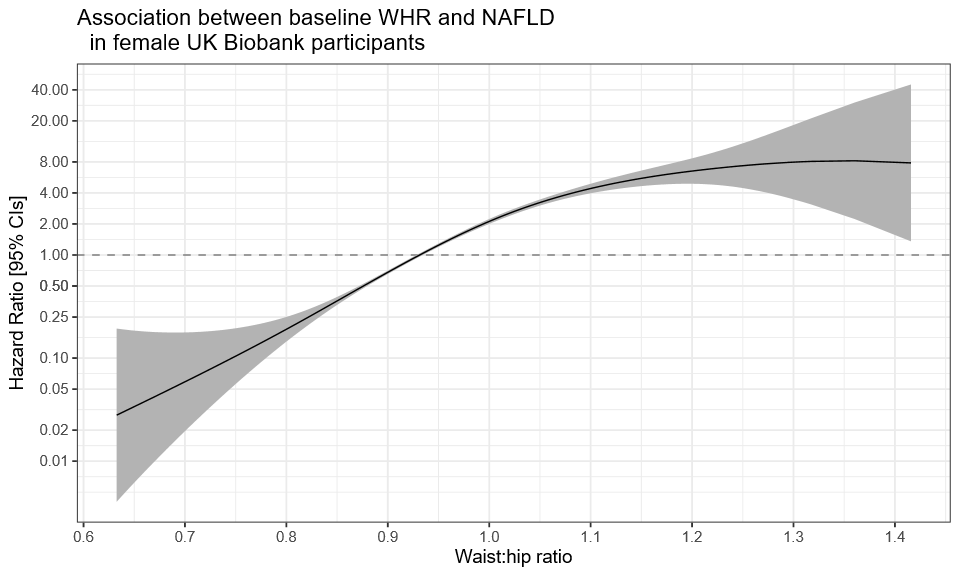


Spline point regression plots in female UK Biobank participants (n=244,409). Graphs a) = liver fibrosis/cirrhosis, b) = NAFLD. Each model was adjusted for age. Abbreviations: CI, confidence interval; WHR, waist-to-hip ratio; NAFLD, non-alcoholic fatty liver disease.

**References**

1. Companion document for serum biomarker data. UK Biobank. November 3, 2019. Accessed April 29, 2024. http://biobank.ctsu.ox.ac.%20uk/crystal/refer.cgi?id=1227

2. Sheard S, Nicholls R, Froggatt J. Haematology Data Companion Document. *UK Biobank* . Published online October 24, 2017. Accessed May 16, 2024. https://biobank.ndph.ox.ac.uk/ukb/ukb/docs/haematology.pdf

3. Sterling RK, Lissen E, Clumeck N, et al. Development of a simple noninvasive index to predict significant fibrosis in patients with HIV/HCV coinfection. *Hepatology*. 2006;43(6):1317-1325. doi:10.1002/hep.21178

4. Tierney A, Fry D, Almond R, Gordon M, Moffat S. UK Biomarker Enhancement Project: Companion Document to Accompany HbA1cBiomarker Data. Published online 2018. Accessed May 14, 2024. https://biobank.ndph.ox.ac.uk/showcase/showcase/docs/serum_hb1ac.pdf

5. Townsend P, Philmore P, Alastair B. Health and deprivation: inequality and the North. *Taylor & Francis*. 2023;8.

6. Craig CL, Marshall AL, Sjöström M, et al. International Physical Activity Questionnaire: 12-Country Reliability and Validity. *Med Sci Sports Exerc*. 2003;35(8):1381-1395. doi:10.1249/01.MSS.0000078924.61453.FB

7. Cassidy S, Chau JY, Catt M, Bauman A, Trenell MI. Cross-sectional study of diet, physical activity, television viewing and sleep duration in 233 110 adults from the UK Biobank; the behavioural phenotype of cardiovascular disease and type 2 diabetes. *BMJ Open*. 2016;6(3):e010038. doi:10.1136/bmjopen-2015-010038

8. Atkins JL, Pilling LC, Masoli JAH, et al. Association of Hemochromatosis *HFE* p.C282Y Homozygosity With Hepatic Malignancy. *JAMA*. 2020;324(20):2048. doi:10.1001/jama.2020.21566

9. Alexander M, Loomis AK, Fairburn-Beech J, et al. Real-world data reveal a diagnostic gap in non-alcoholic fatty liver disease. *BMC Med*. 2018;16(1):130. doi:10.1186/s12916-018-1103-x

10. Murrin O, Mounier N, Voller B, et al. A systematic analysis of the contribution of genetics to multimorbidity and comparisons with primary care data. *MedRxiv* . Published online May 13, 2024.

11. National Academies of Sciences E and M. *Using Population Descriptors in Genetics and Genomics Research: A New Framework for an Evolving Field*. National Academies Press; 2023. doi:10.17226/26902

12. Casanova F, Tian Q, Atkins JL, et al. Iron and risk of dementia: Mendelian randomisation analysis in UK Biobank. *J Med Genet*. Published online January 8, 2024:jmg-2023-109295. doi:10.1136/jmg-2023-109295
